# Moralized Rationality: Relying on Logic and Evidence in the Formation and Evaluation of Belief Can Be Seen as a Moral Issue

**DOI:** 10.1371/journal.pone.0166332

**Published:** 2016-11-16

**Authors:** Tomas Ståhl, Maarten P. Zaal, Linda J. Skitka

**Affiliations:** 1 Department of Psychology, University of Illinois at Chicago, Chicago, Illinois, United States of America; 2 Department of Psychology, University of Exeter, Exeter, United Kingdom; University of Melbourne, AUSTRALIA

## Abstract

In the present article we demonstrate stable individual differences in the extent to which a reliance on logic and evidence in the formation and evaluation of beliefs is perceived as a moral virtue, and a reliance on less rational processes is perceived as a vice. We refer to this individual difference variable as moralized rationality. Eight studies are reported in which an instrument to measure individual differences in moralized rationality is validated. Results show that the Moralized Rationality Scale (MRS) is internally consistent, and captures something distinct from the personal importance people attach to being rational (Studies 1–3). Furthermore, the MRS has high test-retest reliability (Study 4), is conceptually distinct from frequently used measures of individual differences in moral values, and it is negatively related to common beliefs that are not supported by scientific evidence (Study 5). We further demonstrate that the MRS predicts morally laden reactions, such as a desire for punishment, of people who rely on irrational (vs. rational) ways of forming and evaluating beliefs (Studies 6 and 7). Finally, we show that the MRS uniquely predicts motivation to contribute to a charity that works to prevent the spread of irrational beliefs (Study 8). We conclude that (1) there are stable individual differences in the extent to which people moralize a reliance on rationality in the formation and evaluation of beliefs, (2) that these individual differences do not reduce to the personal importance attached to rationality, and (3) that individual differences in moralized rationality have important motivational and interpersonal consequences.

## Introduction

“It is always better to have no ideas than false ones; to believe nothing, than to believe what is wrong.”

- Thomas Jefferson, *Letter to Rev*. *James Madison Paris*, *July 19 1788*.

“But, above all, let it be considered that what is more wholesome than any particular belief is integrity of belief, and that to avoid looking into the support of any belief from a fear that it may turn out rotten is quite as immoral as it is disadvantageous.”

- Charles Sanders Peirce (1877, p. 15).

Human history is replete with examples of new scientific ideas and observations creating tension with normative beliefs of the day. Despite being backed up by strong evidence, defenders of heliocentrism, the theory of evolution by natural selection, as well as the current scientific consensus that human activity causes global warming have all faced ferocious resistance against their ideas [1, 2, 3]. The heat and persistence with which traditional beliefs are defended against scientific conclusions is thought to stem from the fact that those beliefs are closely tied to people’s core moral values [4, 5, 6, 7]. Opinions grounded in moral conviction are different from equally strong but amoral opinions, in that they are perceived as “oughts” rather than as personal preferences, and as objectively true and universally applicable to everyone [8, 9, 10, 11], and lead to intolerance towards those that are attitudinally dissimilar [10, 12, 13, 14, 15, 16]. It is therefore not surprising that traditional beliefs grounded in core moral values are defended so fiercely against scientific advancements.

However, it is not only the morally motivated defenders of traditional beliefs that have been characterized as intolerant in these debates. Advocates of science have also been accused of being strident, angry, and intolerant of their opponents. For example, all of these attributes have frequently been ascribed to the so-called “New Atheists” [17, 18, 19]. At the core of the argument put forth by the New Atheists is the idea that it is important to embrace a scientific world-view, to rely on reason and evidence when evaluating the quality of ideas, and to reject faith-based claims [20, 21, 22, 23]. To the extent that there is any truth to accusations that these promoters of science are angry and intolerant, what do their strong emotions and intolerance stem from? Contrary to the general image of the scientist as objective and value-neutral, we suggest that advocates of science are frequently anything but value-neutral or amoral in their convictions about the superiority of beliefs based on rationality and scientific evidence. More specifically, we suggest that people can come to view it as a moral virtue to form and evaluate attitudes and beliefs based on logical reasoning and evidence, and to view it as a vice to rely on less rational processes, an inclination we refer to as *moralized rationality*.

The purpose of the present research is to investigate whether individuals differ in the extent to which they moralize a reliance on reason and evidence in the formation and evaluation of belief, and whether moralized rationality can be distinguished from the personal importance people attach to being rational. We also examine whether individual differences in moralized rationality predict intolerance of beliefs that are not based on reason and evidence, as well as intolerance of individuals who hold non-rational beliefs. We report a set of eight studies in which we systematically develop and validate an instrument measuring individual differences in moralized rationality (Studies 1–3), examine its test-retest reliability (Study 4), as well as its convergent and discriminant validity (Study 5). We then investigate whether the scale can be used to predict various morally laden indicators of intolerance towards people who rely on an irrational (vs. rational) way of forming/evaluating beliefs in various different domains (Studies 6 and 7). Finally, we examine whether the moralized rationality scale uniquely predicts motivation to contribute to a charity that works to prevent the spread of irrational beliefs (Study 8). Before we outline the present studies in more detail, however, we first discuss the theoretical and empirical foundations of the moralized rationality construct and elaborate on why we think that individual differences in moralized rationality could help to explain intolerance of people who violate (vs. uphold) rational principles of forming and evaluating beliefs.

### Disputes about the validity of beliefs

Modern society is full of examples of disputes that center on the validity of specific beliefs, and where one position is backed up by logical reasoning and scientific evidence, whereas the other is not. A good example is the longstanding debate about whether creationism/intelligent design constitutes a plausible alternative explanation of the origin of species to the theory of evolution, and as a consequence, whether it should be taught alongside the theory of evolution in biology classes [24]. Other examples are the debates about whether human activity causes global warming [25, 26, 27], whether it is good practice to inoculate children against certain viruses [28], and whether it is safe to eat genetically modified food [29, 30]. Psychological research that has tried to explain these disputes focuses almost exclusively on the psychology of those who go against the scientific consensus. For example, some researchers have suggested that opposition to scientific consensus can be attributed to specific personality factors, such as cognitive inflexibility and a lack of openness to experience [31, 32], or political conservatism [33]. Others have instead argued that everyone is susceptible to motivated reasoning biases, and disagree with scientific evidence whenever the implications of the findings are inconsistent with cherished ideological beliefs and values, regardless of whether those beliefs are liberal or conservative [5, 6, 34].

In contrast to earlier work in this area, the present research focuses on the psychology of those who *defend* the scientific consensus. We argue that the motivation to prioritize or privilege rationality can also be moral, but that this moral value is not well-captured by existing theories in moral psychology [4, 35, 36, 37]. More specifically, we suggest that individuals may vary in the extent to which they view it as a moral imperative to rely on logic and evidence when forming and evaluating beliefs. We further argue that individual differences in moralized rationality should have important implications for how people respond when faced with others who either uphold or violate rational principles of how best to evaluate the validity of beliefs. We elaborate on this point in the section below.

### Moralized rationality

Various different strategies can be used to form and evaluate beliefs [38, 39, 40, 41, 42]. For example, people can opt to hold on to whatever their opinion happens to be at the time -- a strategy that has been referred to as *tenacity* [40]. Beliefs and attitudes acquired and upheld in this way are well rehearsed, rarely questioned, and frequently maintained in an effort to be cognitively consistent [43]. This strategy may also account for the observation that people’s pre-existing ideological positions influence their interpretations and evaluations of new ideology-relevant evidence [5, 34]. People can also rely on the opinions of an *authority*. It is well established that appealing to one’s authority is an effective social influence strategy [44], and it may account for much of the influence of various religious authorities on the beliefs of their followers [45]. Alternatively, people can rely on what feels right. This strategy can be referred to as reliance on *intuition*, and it may account for a broad class of phenomena where incidental emotional states guide judgments and behaviors [38, 46, 47]. Non-systematic routes to forming belief, such as the ones discussed above, can be contrasted with the more systematic strategy used in *science*: a combination of reliance on logical reasoning and empirical observation.

It is clear that people differ in the extent to which they prefer to rely on each of the methods discussed above when they form and evaluate ideas. We suggest, however, that they can also differ in the extent to which they view a reliance on specific methods of forming and evaluating beliefs as a moral imperative. We specifically propose that people differ in the extent to which they view it as a moral virtue to rely on a rational/scientific route when forming and evaluating beliefs and attitudes (i.e., a reliance on logic and evidence), and as a vice to rely on irrational/non-systematic routes of forming and evaluating beliefs (e.g., tenacity, authority, intuition). Importantly, our claim that a commitment to rationality can be moral in nature implies that it goes beyond a personal preference to be rational [16, 48]. Because moral values and attitudes are perceived as objectively true, and universally applicable “oughts” [10], people who moralize rationality should not only consider it a moral imperative for themselves, but also think that others ought to rely on rational (vs. irrational) routes when forming or evaluating their beliefs. Indeed, because morally grounded differences of opinion lead to intolerance [16, 48], any moral significance ascribed to a reliance on logic and evidence when forming and evaluating beliefs should lead to intolerance of those who rely on less rational processes. We therefore expect individuals who moralize rationality to be inclined to evaluate the relative rationality of other people’s arguments and beliefs in moral terms. Specifically, we expect individuals who moralize rationality to judge people who speak or behave in ways that violate rational principles of forming and evaluating beliefs to be less moral than those who speak or behave in ways that uphold those principles. An inclination to moralize rationality should therefore lead to a less accepting stance towards people who violate rational principles of forming and evaluating beliefs [12, 16, 49].

Finally, goals that have perceived moral implications are ascribed particular importance [50, 51, 52]. As stated earlier, moral standpoints are also perceived as objectively true, and universally applicable “oughts”, indicating that people who hold a certain moral standpoint believe that everyone should adhere to it. As a consequence, moral values and attitudes are particularly strong predictors of people’s societal and political engagement, activism, and support for groups that personify their moral values and attitudes [53, 54, 55, 56, 57, 58, 59]. We therefore expect those who moralize rationality to be particularly willing to contribute to activism that serves to prevent the spread of irrational beliefs.

### Overview of Studies

In eight studies we investigate individual differences in the moralization of a rational approach to the formation and evaluation of beliefs (moralized rationality). We expected to find individual differences in the extent to which people see rationality as a moral virtue, and irrationality as a vice. Furthermore, based on earlier research on moral values and attitudes we expected moralized rationality to be stable over time, and clearly distinct from the personal importance people attach to rationality [16], as well as from other moral values that have been identified in the literature [53]. In addition, we expected individual differences in moralized rationality to be uniquely predictive of various indicators of intolerance for others’ irrational belief. Specifically, we expected moralized rationality to predict negative moral judgments, moral character evaluations, social distancing, negative moral emotions, and desire to punish people who rely on irrational processes of forming and evaluating beliefs. Finally, we expected individual differences in moralized rationality to uniquely predict willingness to contribute to a charity that serves to prevent the spread of irrational beliefs.

In Study 1 we develop a large pool of items to measure individual differences in moralized rationality, explore their underlying latent structure, and empirically select the most suitable items. In Studies 2 and 3 we examine the underlying factor structure of the selected items more rigorously using confirmatory factor analyses, and in Study 4 we evaluate the test-retest reliability of the scale. In Study 5 we examine the scale’s convergent and discriminant validity. Of particular importance, we investigate whether the moralized rationality scale is conceptually distinct from various existing measures of moral values and inclinations [53, 60]. Once the construct validity of the moralized rationality scale has been established, we turn to its predictive validity in Studies 6–8.

For 7 out of the 8 studies presented in this article, participants were recruited from Amazon’s Mechanical Turk (MTurk). MTurk samples are considerably more demographically diverse than typical university student samples, and studies suggest that data obtained from MTurk samples is at least as reliable as data obtained from student samples [61, 62]. In the final study we examined the predictive validity of the moralized rationality scale in an entirely different sample (i.e., a diverse sample of university students, faculty, and staff).

## Study 1: Exploratory Factor Analysis and Item Selection

The goal of Study 1 was to generate and evaluate a pool of items designed to measure the extent to which individuals view a rational approach to the formation and evaluation of beliefs (i.e., one based on logic and evidence) as a moral virtue. Because measures of the moralization of specific issues can easily be confounded with the perceived non-moral importance of the same issue [10], we also generated and evaluated a set of items measuring the personal (non-moral) importance individuals attach to formation and evaluation of belief on rational grounds. Although the non-moral importance of rationality was not the focal variable in the present studies, we took care to apply the same high standards to the validation of the Importance of Rationality (IR) scale as we did to the Moralized Rationality (MR) scale. This approach enabled us (1) to test the degree to which moralized rationality is distinct from the personal importance people attach to rationality, and (2) to effectively control for the personal importance attached to rationality in our analyses of the effects of moralized rationality, thereby allowing us to establish the unique contributions of MR and to rule out perceived (non-moral) importance of rationality as an alternative explanation of the results [16].

As a first step in the scale construction process, we generated a set of items designed to measure the extent to which individuals see a reliance on rationality in the formation and evaluation of belief (1) as a moral issue, and (2) as personally important to them. We took care to balance the number of items presenting rationality as a moral virtue with the number of items presenting irrationality as a vice. Based on earlier theoretical work [39, 40], we generated a number of items that measured the moralization (and perceived importance) of the reliance on rationality in the formation and evaluation of belief compared to the reliance on less rational methods (i.e., tenacity, tradition, authority, and intuition). The total number of items was then brought to 44 through mutual discussion. Twenty-two of these items measured moralized rationality, the other 22 measured the perceived personal importance of rationality (for a list of all items, see S1 Text). Study 1 was designed to serve two purposes. First, this study served as a first exploratory test of the underlying latent structure of our set of items. We expected the moralization of rationality and the perceived personal importance of rationality to be correlated, but nonetheless distinct. Second, Study 1 was designed to help us reduce the number of items in each scale by allowing us to select those items that most closely correspond to the latent variables underlying both scales.

### Method

#### Sample, procedure and materials

This study was approved by the Ethics Committee of the Social, Environmental, and Organizational Psychology Research Group (S.E.O.R.G.) at University of Exeter. Three hundred US residents were recruited from Amazon’s Mechanical Turk. They received $1.50 for their participation that lasted approximately 15 minutes. Fifty-eight percent were male and 42% female (*M*_age_ = 33.59, *SD* = 10.39). Seventy-eight percent self-identified as Caucasian, 9% as Asian American, 6% as African American, 5% as Hispanic/Latino, and 1% as other.

Upon completing the informed consent form, participants filled out an online survey that consisted of the 44 items intended to measure Moralized Rationality and the Importance of Rationality, as well as the Moral Foundations Questionnaire (MFQ) including the foundation of liberty [53, 63]. We included the MFQ in this study, and in four additional studies reported in this article, for the purpose of evaluating the discriminant validity of the moralized rationality scale. For reasons of brevity, we report all analyses related to the MFQ when we examine the convergent and discriminant validity of the moralized rationality scale (i.e., in Study 5). The MR and IR items were mixed together and the order in which they were presented was randomized. The order of the MR/IR items and the MFQ was counterbalanced. Upon completion of the survey, participants were thanked and paid for their participation.

### Results and discussion

We excluded participants who took part more than once (*n* = 6) as well as extremely fast responders (*n* = 11), resulting in a final sample of 283 participants. In all studies reported in this article, extremely fast responders were excluded prior to conducting any data analyses. All cut offs were based on pilot testing. It should also be noted that including omitted participants in the analyses did not significantly alter the results in any of the studies.

The MR and IR items were analyzed with exploratory factor analysis using the Maximum Likelihood method [64]. Because we expected importance of rationality and moralized rationality to be correlated, we opted for an oblique rotation method (direct oblimin). Inspection of the scree plot and factor loadings revealed a two factor solution that accounted for 41% of the variance, and that clearly differentiated between the items measuring MR (Factor 1) and those measuring IR (Factor 2). As expected, the two factors were positively correlated, *r* = .30.

To select only the most suitable items for our MR and IR scales, we used the following criteria. We selected items that had a factor loading of .65 or higher on one factor, and that did not have a factor loading higher than .3 on the alternative factor. A total of 17 items fit these criteria, resulting in a preliminary 10-item Moralized Rationality Scale (MRS, α = .92), and a 7-item Importance of Rationality Scale (IRS, α = .89). In Study 2 we tested the two-factor structure of the MRS and IRS using confirmatory factor analysis on a different sample.

## Study 2: Confirmatory Factor Analysis

### Method

#### Sample, procedure, and materials

This study was approved by the Ethics Committee of the Social, Environmental, and Organizational Psychology Research Group (S.E.O.R.G.) at University of Exeter. Five hundred US residents were recruited from Amazon’s Mechanical Turk. However, four individuals who completed the survey did not click through to the very last page on MTurk.com. These four assignments were therefore not registered as completed, and automatically republished on MTurk.com, resulting in a final sample size of 504. (59% men, 41% women, *M*_age_ = 34.76 *SD* = 11.75). Participants received $0.60 for their time (approximately 5 minutes). Eighty percent self-identified as Caucasian, 7% as Asian, 6% as African American, 6% as Hispanic or Latino, 0.4% as Native American, and 2% as other. After signing the informed consent form, participants completed the 10-item MRS and the 7-item IRS. The items of the MRS and IRS were mixed together and presented in random order.

### Results and discussion

We excluded participants who took part more than once (*n* = 16) as well as extremely fast responders (*n* = 10), resulting in a final sample of 479 participants. We fitted the proposed two-factor model, with the MRS and IRS items loading on separate factors (no cross-factor loadings were allowed), and allowed the two factors to correlate. The error terms of the two negatively phrased MRS items were also allowed to correlate to correct for a method effect [65]. We consider the proposed model to fit the data well when SRMS ≤ .08, RMSEA ≤ .06, and CFI and NFI ≥ .95, and reasonably well when .08 > SRMS < .10, .06 < RMSEA < .08, and .90 < CFI and NFI < .95 [66].

The results showed that this model fit the data reasonably well, although some of the fit statistics failed to reach recommended levels [66], χ^2^(117) = 506, *p* < .001, RMSEA = .08, CFI = .89, NFI = .86, SRMR = .08. Inspection of the modification indices revealed that model fit could be substantially improved by removing two items, one from the IRS and one from the MRS. The IRS item “Rationality and evidence are not important to me (R)” exhibited a floor effect (over 50% of the sample indicated fully disagreeing with it). The MRS item “Relying on logic and evidence when forming beliefs, rather than on tradition, is a moral virtue” lowered model fit only because it had a high residual correlation with one of the other MRS items (“Being skeptical about claims that are not backed up by evidence is a moral virtue”). After deletion of these two items, the fit of the proposed two-factor model was acceptable, χ^2^(117) = 338, *p* < .001, RMSEA = .076, CFI = .92, NFI = .89, SRMR = .07, with all items loading strongly on their corresponding factor (all *p*’s < .001). The MR and IR factors were significantly correlated, *r* = .23, *p* < .001.

We then compared this model to the most plausible alternative, a model in which all items of the MRS and IRS were explained by a single latent factor. The error terms of the two negatively phrased MRS items were once again allowed to correlate to correct for a method effect [65]. The results showed that this alternative model did not fit the data well, χ^2^(89) = 1195, *p* < .001, RMSEA = .16, CFI = .64, NFI = .62, SRMR = .14. A Chi-square difference tests showed that the two-factor model fit the data significantly better than the one-factor alternative, Δχ^2^(1) = 857, *p* = < .001.

The results of Study 2 thus show that the extent to which individuals moralize rationality can be clearly distinguished from the extent to which they place personal importance on rationality. In addition, the acceptable fit of the two-factor model indicates that scores on the MRS and IRS are each driven by a single latent variable. To achieve such acceptable fit, however, we had to make post hoc changes to the MRS and IRS. The results of Study 2 therefore cannot be considered fully confirmatory. To address this issue, we test the latent structure of the modified 9-item MRS and 6-item IRS in Study 3.

## Study 3: Confirmatory Factor Analysis II

### Method

#### Sample, procedure, and materials

This study was approved by the Ethics Committee of the Social, Environmental, and Organizational Psychology Research Group (S.E.O.R.G.) at University of Exeter. Four hundred US residents were recruited from Amazon’s Mechanical Turk. One participant failed to click through to the last page, which resulted in a final sample of 401 (51% men, 49% women, *M*_age_ = 33.91 *SD* = 10.77). Participants took part in a 20 minute testing session of which the present 5-minute study was the first part. They received $2.00 for their participation in the full session. Seventy-seven percent self-identified as Caucasian, 8% as Asian, 7% as African American, 5% as Hispanic or Latino, 1% as Native American, and 2% as other.

Participants completed the 9-item MRS, the 6-item IRS, as well as the Moral Foundations Questionnaire [53, 63]. As in Studies 1 and 2, the MRS and IRS items were mixed and presented in random order. In contrast to Study 1, the order in which the MRS/IRS and MFQ were presented was not counterbalanced (the MFQ was administered first).

### Results and discussion

As in Studies 1 and 2, we filtered out participants who took part more than once (*n* = 18) as well as extremely fast responders (*n* = 22), resulting in a final sample of 361. As in Study 2, we fitted the proposed two-factor model to the data, with the MRS and IRS items loading on different factors, and allowed the two factors to correlate. Finally, as in Study 2, we allowed the error terms of the two negatively phrased MRS items to correlate in order to correct for a method effect [65].

The results showed that this model fit the data well, χ^2^(88) = 214, *p* < .001, RMSEA = .06, CFI = .94, NFI = .90, SRMR = .06, with all items loading strongly on their corresponding factor (*p* < .001). The two factors showed a significant positive correlation, *r* = .32, *p* < .001.

As in Study 2, the alternative model in which all MRS and IRS items loaded on a single factor did not fit the data well, χ^2^(89) = 884, *p* < .001, RMSEA = .16, CFI = .60, NFI = .58, SRMR = .15. A Chi-square difference test showed that the two-factor model fit the data significantly better than the one-factor alternative, Δχ^2^(1) = 670, *p* = < .001.

The results of Study 3 replicate the results of Study 2 and show that the extent to which individuals moralize rationality can be clearly distinguished from the extent to which they see rationality as personally important. In addition, Study 3 showed good fit of the two-factor model that was predicted to underlie scores on the MRS and IRS. This indicates that, as expected, scores on the MRS and IRS are each driven by different latent variables. The means, standard deviations, reliability coefficients, and correlations of the 9-item MRS and the 6-item IRS in each of the studies presented in this article are reported in Table 1. All of the items from the final version of the MRS and the IRS are presented in Table 2, along with their loadings on the MR and IR factors, respectively (participants from all of the studies reported in the present research [except Study 4] were included in this factor analysis, *N* = 2452).

**Table 1:** Descriptive Statistics. Means, Standard Deviations, Reliabilities, and Correlations of the 9-item Moralized Rationality Scale and the 6-item Importance of Rationality Scale (Studies 1–7).

		*M*	*SD*	α	*r*_*MRS-IRS*_
Study 1 (*N* = 283)					
	MRS	3.80	1.34	.91	.32***
	IRS	5.70	1.06	.88	
Study 2 (*N* = 479)					
	MRS	3.78	1.17	.88	.32***
	IRS	5.66	0.98	.86	
Study 3 (*N* = 361)					
	MRS	3.71	1.14	.86	.23***
	IRS	5.67	0.93	.84	
Study 4 (time 2, *N* = 198)					
	MRS	3.77	1.35	.92	.34***
	IRS	5.75	1.04	.88	
Study 5 (*N* = 183)					
	MRS	3.58	1.22	.89	.32***
	IRS	5.63	1.00	.85	
Study 6 (*N* = 574)					
	MRS	3.70	1.25	.90	.33***
	IRS	5.64	0.98	.85	
Study 7 (*N* = 252)					
	MRS	3.78	1.27	.91	.34***
	IRS	5.68	0.91	.84	
Study 8 (*N* = 311)					
	MRS	3.76	1.01	.79	.42***
	IRS	5.61	0.90	.79	

*** *p* < .001

**Table 2:** Factor Loadings. Factor Loadings Based on an Exploratory Factor Analysis Using the Maximum Likelihood Method with Direct Oblimin Rotation of the Items of the Moralized Rationality and Importance of Rationality Scales (Data from Studies 1, 2, 3, 5, 6, 7, and 8. *N* = 2452).

Item	Factor loadings	
	MR	IR
Moralized Rationality		
1. Being skeptical about claims that are not backed up by evidence is a moral virtue.	.**61**	.16
2. Holding on to beliefs when there is substantial evidence against them is immoral.	**.76**	.04
3. It is morally wrong to trust your intuitions without rationally examining them.	**.68**	.00
4. It is morally wrong to rely on anything else other than logic and evidence when deciding what is true and what is not true.	**.72**	.04
5. It is a moral imperative that people can justify their beliefs using rational arguments and evidence.	**.68**	.15
6. It is immoral to hold irrational beliefs.	**.77**	-.05
7. A person’s moral authority depends on their rationality.	**.64**	.04
8. A person’s morality is in no way determined by their rationality. (R)	**-.53**	.04
9. Whether a person can be convinced by reason and evidence is in no way indicative of their morality. (R)	**-.57**	.13
Importance of Rationality		
1. It is important to me personally to be skeptical about claims that are not backed up by evidence.	.05	**.72**
2. It is important to me personally to remain rational and levelheaded even in heated arguments.	-.09	**.59**
3. It is important to me personally to examine traditionally held beliefs using logic and evidence.	.04	**.77**
4. It is important to me personally that I can justify my beliefs using rational arguments and evidence.	.10	**.74**
5. It is important to me personally to critically examine my long-held beliefs.	.02	**.63**
6. It is important to me personally to be a rational person.	-.02	**.69**

In Studies 1 through 3 we developed measures of moralized rationality and the importance of rationality. The results of these studies showed that moralized rationality can be clearly distinguished from the (amoral) importance of rationality, that the measures we developed have high internal consistency, and have the hypothesized latent structure. In Study 4 we examined the test-retest reliability of the MRS.

## Study 4: Test-Retest Reliability

### Method

#### Sample, procedure, and materials

This study was approved by the Ethics Committee of the Social, Environmental, and Organizational Psychology Research Group (S.E.O.R.G.) at University of Exeter. Contrary to what is suggested by the ordering of the studies presented in this paper, Study 4 was the third-to-last of the present set of studies to be conducted, taking place approximately four months after Study 1 and two months after Study 6. To enable us to determine the test-retest reliability of the MRS, we contacted 300 individuals who we had randomly selected from the data of Studies 1 and 6 (150 participants from each study) and invited them to take part in a 5 minute follow-up study. One hundred and ninety eight individuals (96 from Study 1, 102 from Study 6, 66% of the total number that were contacted) took part in exchange for $1.50 (61.6% men, 39.4% women, *M*_age_ = 36.05, *SD* = 11.91). Participants were contacted through Amazon Web Services, using only their MTurk identification codes. This procedure ensured that all participants remained anonymous.

As in Studies 1 through 3, the items of the MRS (*α =* .92, *M* = 3.77, *SD* = 1.35) and IRS (*α =* .89, *M* = 5.75, *SD* = 1.04) were mixed together and presented in random order. Individuals who took part in the follow-up study did not systematically differ from individuals who did not in terms of their average scores on the MRS, *t*(299) = -0.41, *p* = .68 and IRS, *t*(299) = 0.76, *p* = .45.

### Results and discussion

To determine the test-retest reliability of the Moralized Rationality Scale, original MRS scores (from Studies 1 and 6) were correlated with the MRS scores obtained in the follow-up study. The results showed that MRS scores were highly stable over time, *r*(194) = .77, *p* < .001. We then tested whether original MRS scores were more highly correlated with follow-up MRS scores among individuals who were re-contacted after taking part in Study 6 (a two month time interval between the two measurements) than among individuals who were re-contacted after taking part in Study 1 (a four month time interval between the two measurements). The results indicated that Study 6 MRS scores (*r*[98] = .75, *p* < .001) were not more predictive of follow-up MRS scores than Study 1 MRS scores (*r*[96] = .79, *p* < .001), *p* = .69. The results thus show that, not only are MRS scores highly stable over time, but their stability over time is not affected by time interval (2 months vs. 4 months).

As a side note, the IRS also demonstrated high overall test-retest reliability (*r* = .79). Moreover, the test-retest reliability of the IRS was unaffected by time interval (*p* = .96). In Study 5, we turn to investigate the convergent and discriminant validity of the MRS.

## Study 5: Convergent and Discriminant Validity of the MRS

We examined the convergent and discriminant validity of the MRS in several different ways. First, we investigated the relation between the MRS and strength of belief in science [67], and paranormal and religious beliefs [68, 69]. Because the MRS was developed to measure the extent to which individuals moralize a reliance on *rational* (vs. *irrational*) methods of forming and evaluating belief, we expected it to be positively related to belief in science, as well as to our own measure of importance of rationality (IRS), and to be negatively related to paranormal beliefs and religiosity. Second, we investigated whether the MRS taps into a *moral* construct, and one that is not captured by any existing measures in moral psychology. Inspired by the moral identity scale [70], we developed measures to assess the extent to which various different traits are perceived as prototypical of a moral role model and of an immoral person. To the extent that the MRS taps into a unique moral value, individuals who moralize rationality should view rationality-oriented traits as more prototypical of a moral role model, and view irrationality-oriented traits as more prototypical of an immoral person. By contrast, the MRS should not uniquely predict the perceived prototypicality of traits (of a moral role model vs. immoral person) that are unrelated to the rational formation and evaluation of beliefs.

We also sought to investigate moralized rationality’s relation to utilitarianism [60, 71]. A utilitarian approach to morality implies that the perceived morality of an action is determined by its consequences [71]. Thus, from a utilitarian perspective, a harmful act is justified to the extent that it minimizes the total harm done. Utilitarianism is typically contrasted with deontology, according to which the morality of an action is intrinsic to the action itself [72]. Doing harm is never justifiable based on deontological principles, because it is intrinsically immoral. We can think of no plausible reason why moralized rationality should reduce to a general inclination to rely on deontological moral principles. However, it could be argued that individuals who moralize rationality do so because they expect irrational processes of evaluating beliefs to have harmful consequences [35, 73]. In other words, moralized rationality could be just one example of a more general inclination to evaluate the morality of actions based on their consequences. To examine this issue, we included the scenarios developed by Conway and Gawronski [60] to measure utilitarian and deontological inclinations in moral dilemmas. If moralized rationality boils down to utilitarianism (or deontology), the MRS should be closely related to utilitarian (or deontological) inclinations. However, we argue that individuals high in moralized rationality view irrational belief as immoral because it is not based on reason and evidence, not because it is potentially harmful. We therefore did not anticipate a strong correlation between the MRS and utilitarian inclinations.

To further establish the discriminant validity of the MRS, we also investigated how it relates to the moral foundations proposed by Haidt and his colleagues [53]. Research on moral foundations theory initially identified five different moral values, or “moral foundations”, on which people rely to a varying extent when they evaluate the morality of specific actions [4]. The Care/harm foundation is thought to serve the protection of vulnerable individuals, and is triggered by signs of suffering. In a similar vein, the Fairness/cheating foundation serves to prevent exploitation, and is therefore highly sensitive to unfair treatment. The Loyalty/betrayal foundation is responsive to signs of disloyalty to the group, and thought to serve the purpose of forming and maintaining strong coalitions. The Authority/subversion foundation serves to preserve the integrity of established status hierarchies within the group, and is therefore sensitive to signs of disrespect of legitimate authorities. Finally, the Sanctity/degradation foundation is triggered by acts that are perceived as unnatural or disgusting, and it is thought of as an adaptation to the risk of exposure to various pathogens. More recently, concerns about individual freedom have been proposed as a potential sixth moral foundation (i.e., Liberty/oppression; [63]). We expected moralized rationality to be conceptually distinct from all of the moral foundations. Therefore, weak correlations were expected between the MRS and endorsement of Care/harm, Fairness/cheating, Loyalty/betrayal, Authority/subversion, Sanctity/degradation, and Liberty/oppression. As discussed above, one might suspect that the inclination to moralize rationality could be attributable to utilitarian harm concerns [35, 73]. It was therefore of particular importance to rule out a strong relationship between the MRS and endorsement of the Care/harm foundation. Due to the length of this survey, and particularly the battery of scenarios needed to measure utilitarian and deontological inclinations, we did not assess endorsement of the moral foundations in Study 5. However, measures of the moral foundations were included in Study 1, Study 3, Study 6, Study 7, as well as in Study 8 (Liberty/oppression was not included in Study 8). Because this study focuses on the construct validity of the MRS, we opt to discuss all of the results related to the moral foundations here.

We also wished to investigate the extent to which the MRS is sensitive to socially desirable responding. To this end, we included the Balanced Inventory of Desirable Responding [74], which measures individuals’ tendency to engage in impression management and self-deceptive enhancement. Finally, political orientation, as well as several demographic variables were measured for exploratory purposes.

### Method

#### Sample

This study was approved by the Ethics Committee of the Social, Environmental, and Organizational Psychology Research Group (S.E.O.R.G.) at University of Exeter. Two hundred US residents were recruited from Amazon Mechanical Turk. Two participants failed to click through to the very last page of the survey, which resulted in a final sample of 202. The study lasted approximately twenty minutes, for which participants received a $2 payment. Fifty-seven percent were male and 43% were female (*M*_age_ = 33.83, *SD* = 10.90). Seventy-seven percent self-identified as Caucasian, 7% as Asian American, 7% as African American, 6% as Hispanic/Latino, 1% as Native American, and 1% as other.

#### Procedure and materials

Upon giving informed consent, participants took part in an online survey. Below is a description of all the measures included, listed in the order they were presented to participants.

To measure *paranormal beliefs*, we used the six-item paranormal scale [68]. Participants were asked to indicate to what extent they believe in extra-sensory perception, psychics, astrology, reincarnation, precognition, and the possibility of communicating with the dead (1 = *Definitely not*, 4 = *Yes*, *definitely*, *α =* .91).

Participants were also asked to think about a person that they consider as a moral role model. They were then asked to indicate to what extent thirteen different traits characterize a moral role model (1 = *not at all*, 7 = *very much*). Most relevant for the present purposes, four of the traits were related to rationality (rational, analytical, logical, skeptical). The remaining nine traits (caring, compassionate, fair, friendly, generous, helpful, hardworking, honest, kind) were taken from the description of a moral person in the introduction to the moral identity scale [70]. Participants were also asked to think of a person that they consider being immoral (the order of the moral role model vs. immoral person questions was counterbalanced). They were then asked to indicate to what extent thirteen different traits characterize an immoral person (1 = *not at all*, 7 = *very much*). Four of the traits were related to irrationality (irrational, uncritical, unreasonable, gullible). The remaining nine traits were the antonyms of the traits used by Aquino and Reed [70] to describe a moral person (cruel, indifferent, unfair, unfriendly, selfish, unhelpful, lazy, dishonest, unkind). Scores on the rationality-related and irrationality-related traits were averaged to create a reliable eight-item *Prototypicality of rationality for morality scale* (α = .79). Scores on the traditional moral traits used by Aquino and Reed [70], as well as their antonyms, were averaged to create a reliable eighteen-item *Prototypicality of traditional moral traits scale* (α = .91).

We also administered the 10-item *Belief in Science Scale* developed by Farias and colleagues [67]. Examples of items are: “Science tells us everything there is to know about what reality consists of", and "All the tasks human beings face are soluble by science" (1 = *Strongly disagree*, 6 = *Strongly agree*, α = .96). Participants were also presented with the *MRS and IRS*. The items from the MRS (α = .89) and the IRS (α = .85) were mixed together, and presented in a random order.

To measure *socially desirable responding*, we used the 40-item Balanced Inventory of Desirable Responding [74]. This inventory consists of two 20-item subscales, measuring Impression management (IM, α = .86) and Self-deceptive enhancement (SDE, α = .82). An example of an IM item is: “When I hear people talking privately, I avoid listening”. An example of an SDE item is: “I never regret my decisions” (1 = *not at all true*, 7 = *very true*).

We administered the 20 scenario items developed by Conway and Gawronski [60] to measure *utilitarian* and *deontological inclinations*. Notably, these scenario items do not consistently pit utilitarian and deontological inclinations against each other. Instead, only half of the items pit utilitarian principles against deontological principles (incongruent items), whereas these principles are aligned on the remaining items (congruent items). As a consequence, process dissociation [75] can be used to generate separate (and typically independent) measures of utilitarian and deontological inclinations [60].

*Political orientation* on social and economic issues was measured with two items: “How would you describe your political orientation with regard to social/economic issues?” (1 = *Very liberal*, 7 = *Very conservative*). To measure *religiosity* we used three items from the Santa Clara Strength of Religiosity [69] scale (α = .97). An example item is: “My religious faith is extremely important to me” (1 = *not at all*, 5 = *very much*). Finally, we measured participants’ gender, age, and level of education. After that, participants were thanked and paid for their participation.

### Results

#### Demographics, political orientation, and socially desirable responding

As in the previous studies, we filtered out participants who took part more than once (*n* = 10) as well as extremely fast responders (*n* = 8), resulting in a final sample of 184. The only demographic variable that was significantly related to scores on the MRS was gender. Men (*M* = 3.81, *SD* = 1.24) scored higher on the MRS than did women (*M* = 3.29, *SD* = 1.12), *t*(181) = 2.94, *p* = .004. Age (*r* = -.08, *p* = .29), level of education (*r* = .06, *p* = .44), and political orientation on social (*r* = -.07, *p* = .33) as well as economic issues (*r* = -.08, *p* = .27), were all unrelated to the MRS. People who scored high (vs. low) on the MRS were *less* inclined to engage in impression management (*r* = -.21, *p* = .005), whereas there was no relationship between the MRS and self-deceptive enhancement (*r* = -.09, *p* = .22).

Three notable differences in the associations between demographic variables were observed between the MRS and IRS. First, unlike the MRS, high scores on the IRS were associated with being more liberal on social issues (*r* = -.38, *p* < .001). Second, and also unlike the MRS, people who scored high (vs. low) on the IRS were more inclined to engage in self-deceptive enhancement (*r* = .24, *p* = .001). Finally, unlike the MRS, there was no relationship between the IRS and impression management (*r* = .07, *p* = .36). In conclusion, men were somewhat more inclined to moralize rationality than women. In addition, people who moralize rationality appear to be less inclined to engage in certain kinds of socially desirable responding (impression management), whereas people who view rationality as important to them personally are not. In fact, the results suggest that people who view rationality as important to them personally are particularly inclined to engage in self-deceptive enhancement. Finally, although the MRS is unrelated to political orientation, people who score high (vs. low) on the IRS are typically more liberal on social issues.

#### Convergent and Discriminant Validity

All zero-order correlations are presented in Table 3. As expected, the MRS was positively related to the measures tapping into the value people attach to rational processes of evaluating beliefs (IRS, belief in science), and negatively related to measures of attitudes and beliefs that are not formed based on reason and evidence (paranormal beliefs, religiosity). Turning to how the MRS relates to measures of morality, the results suggest that moralized rationality does not reduce to utilitarianism. In fact, there was no relationship at all between the MRS and utilitarian inclinations. Moralized rationality was *negatively* related to deontological inclinations. However, the MRS only shared approximately 3.5% of variance with deontology. Thus, the results suggest that the MRS is conceptually distinct from utilitarian as well as deontological inclinations.

**Table 3:** Zero-order Correlations (Study 5).

Variable	1	2	3	4	5	6	7	8	9	10	11
1 MRS	-										
2 IRS	.32*	-									
3 Belief in science	.34*	.48*	-								
4 Paranormal scale	-.18*	-.25*	-.30*	-							
5 Religiosity	-.17*	-.40*	-.76*	.25*	-						
6 Prototypicality of rationality for morality	.37*	.24*	.21*	-.03	-.06	-					
7 Prototypicality of traditional moral traits	.14†	.14†	.03	-.03	.08	.53*	-				
8 Utilitarianism	-.09	.15*	-.06	-.13†	-.08	-.24*	.05	-			
9 Deontology	-.19*	-.07	-.30*	.10	.31*	-.09	-.03	-.15*	-		
10 IM	-.20*	.07	-.11	-.05	.14†	.08	.20*	.04	.26*	-	
11 SDE	-.09	.21*	.04	.01	.00	-.02	.01	.03	.01	.49*	-

* *p* < .05;

^†^ *p* < .10

As expected, individuals who scored high (vs. low) on the MRS were more inclined to view rationality as prototypical of a moral role model, and irrationality as prototypical of an immoral person. By contrast, the MRS was only marginally positively related to viewing traditional moral traits (or their antonyms) as prototypical of a moral role model (or an immoral person). As can be seen in Table 3, however, the perceived prototypicality of (ir)rationality was also positively related to the IRS, as well as to the perceived prototypicality of traditional moral traits in people’s judgments of moral (immoral) others. We therefore needed to determine whether the MRS *uniquely* predicted the perceived prototypicality of (ir)rationality for a moral role model (immoral person), while controlling for IRS, as well as for the prototypicality of traditional moral traits for a moral role model (immoral person). We therefore regressed the prototypicality of rationality for morality scale on the MRS, IRS, and the prototypicality of traditional moral traits scale. The model explained a significant amount of variance, *F*(3, 179) = 34.45, *p* < .001, ΔR^2^ = .37. Viewing traditional moral (immoral) traits as prototypical for a moral (immoral) person predicted the prototypicality of rationality for morality scale, β = .48, *t*(179) = 7.86, *p* < .001. More importantly for the present purposes, so did the MRS, β = .28, *t*(179) = 4.44, *p* < .001. As expected, the IRS did not predict the prototypicality of rationality for morality scale when we controlled for MRS, β = .07, *t*(179) = 1.14, *p* = .26. These results suggest that individuals who moralize rationality are uniquely inclined to view rationality as prototypical of a moral role model, and to view irrationality as prototypical of an immoral person.

To examine whether the MRS also contributed to the perceived prototypicality of traditional moral traits, we regressed the prototypicality of traditional moral traits scale on the MRS, IRS and the prototypicality of rationality for morality scale. This model explained a significant amount of variance, *F*(3, 179) = 23.19, *p* < .001, ΔR^2^ = .28. The prototypicality of rationality for morality scale came out as a highly significant predictor, β = .54, *t*(179) = 7.86, *p* < .001. As expected, the MRS (β = -.09, *t*(179) = -1.24, *p* = .22), as well as the IRS (β = .06, *t*(179) = .86, *p* = .39) did not predict the prototypicality of traditional moral traits scale. Thus, individuals who score high (vs. low) on the MRS are uniquely inclined to view rationality as a prototypical trait of moral role models, and irrationality as prototypical of immoral people. However, people who score high (vs. low) on the MRS are no more inclined to view traditional moral traits as prototypical of moral role models, or traditional immoral traits as prototypical of immoral people. These results attest to the specificity and precision of the MRS. That is, moralized rationality, as measured by the MRS, does not appear to be confounded with more general conceptualizations of morality [70]. Rather, the MRS specifically measures the extent to which people view it as a moral virtue to rely on reason and evidence when forming and evaluating beliefs, including beliefs about what constitutes a prototypical moral or immoral person.

To summarize, the results of this study suggest that the MRS has high convergent validity. People who score high (vs. low) on the MRS are more inclined to hold attitudes and beliefs associated with valuing logical reasoning and evidence, and they are less inclined to hold attitudes and beliefs that are not supported by logical reasoning or evidence, but instead were formed based on less rational processes. Furthermore, people who score high (vs. low) on the MRS are also inclined to view rationality as a prototypical trait of moral role models, and to view irrationality as a prototypical trait of immoral people. The present study also provided support for the discriminant validity of the MRS. Specifically, we found that the MRS is conceptually distinct from utilitarian as well as deontological inclinations, and that it is unrelated to political orientation, education, and self-deceptive enhancement. The MRS was negatively related to impression management. However, these variables only shared approximately 4% of the variance. Thus, moralized rationality cannot be reduced to a low inclination to engage in impression management. What this negative correlation does suggest, however, is that people who endorse rationality as an important moral value are unlikely to do so for strategic impression management purposes.

Due to the length of the survey, we did not include measures of the *moral foundations* in Study 5. However, we did include measures of the moral foundations in Studies 1 and 3 -- as well as in Studies 6, 7, and 8. Because this study centers on the convergent and discriminant validity of the MRS, we discuss all of these results here. Table 4 displays how the MRS (and IRS) related to all of the moral foundations in each of the five studies. The results suggest that the MRS is conceptually distinct from all of the moral foundations. In all five studies, the MRS was unrelated to Care/harm, Authority/subversion, and Liberty/oppression. Notably, the consistent finding across five studies that the MRS is unrelated to the Care/harm foundation provides additional support for the notion that moralized rationality does not reduce to concerns about harm. The MRS was positively related to Loyalty/betrayal in Study 3, and negatively related to Sanctity/degradation in Study 7. However, because these relationships did not replicate in any of the other four studies, these correlations should be interpreted with great caution. The only significant relationship between the MRS and any of the moral foundations that was consistent across studies, except in Study 7, was a positive relationship with the Fairness/cheating foundation. However, it should be noted that the MRS and the Fairness/cheating foundation consistently shared less than 4% of the variance. Thus, the results suggest that the MRS captures a moral value that is conceptually distinct from all of the moral foundations.

**Table 4:** Zero-order Correlations. Correlations of the 9-item Moralized Rationality Scale and the 6-item Importance of Rationality Scale with Each of the Moral Foundations.

Moral Foundation	Care	Fairness	Loyalty	Authority	Sanctity	Liberty
Study 1 (*N* = 283)						
MRS	.06	.19**	.03	-.07	-.09	.01
IRS	.16**	.36**	-.17**	-.28***	-.33***	.21***
Study 3 (*N* = 363)						
MRS	-.04	.11*	.20***	.08	.09	-.01
IRS	.20***	.34***	-.08	-.11*	-.21***	.21***
Study 6 (*N* = 574)						
MRS	.04	.16***	.02	.00	.00	-.04
IRS	.20***	.34***	-.25***	-.25***	-.27***	.14***
Study 7 (*N* = 253)						
MRS	-.04	.05	-.07	-.09	-.14*	.03
IRS	.19**	.33***	-.22***	-.15*	-.23***	.21**
Study 8 (*N* = 311)						
MRS	.05	.13*	.11	-.04	-.04	-
IRS	.11*	.21***	-.09	-.06	-.17***	-

* *p* < .05;

** *p* < .01;

*** *p* < .001

As can be seen in Table 4, the IRS showed a very different pattern of correlations with the moral foundations than did the MRS. In all studies, the IRS was positively related to Care/harm, Fairness/cheating, and Liberty/oppression. Moreover, the IRS was negatively related to Loyalty/betrayal in three studies, to Authority/subversion in four studies, and to Sanctity/degradation in all five studies. This pattern of correlations is not surprising, in light of the fact that the IRS is correlated with a liberal orientation on social issues [76].

### Discussion and introduction to Studies 7–9: Testing the predictive validity of the MRS

The findings from Study 5, as well as the consistently low correlations between the MRS and all of the moral foundations across five studies, lead us to conclude that the MRS is a valid measure of individual differences in the inclination to moralize rationality. In the final three studies we turn to the predictive validity of the MRS. To the extent that the MRS taps into meaningful individual differences in the inclination to moralize rational ways of forming and evaluating beliefs, it should affect reactions to acts that violate (vs. uphold) rational principles across various different domains. Specifically, we should expect individuals who score high (vs. low) on the MRS to judge acts that violate (vs. uphold) rational principles of forming and evaluating beliefs as less moral. Expressed differently, the MRS should moderate the extent to which people view an act that violates rational principles as less moral than an act that upholds rational principles.

People who moralize rationality should not only respond more strongly to irrational (vs. rational) acts, but also towards the actors themselves. We tested this notion in various different ways. First, it is well established that people assign blame to those who act in immoral ways [46, 77, 78]. Because individuals who score high (vs. low) on moralized rationality should view irrational acts as immoral, they should also assign more blame to a person who acts in a way that violates (vs. upholds) rational principles. Second, because actions have consequences for evaluations of the actor’s character [78], individuals who score high (vs. low) on the MRS, should be more inclined to view someone who acts in an irrational way as a less moral *person* than someone who acts in a rational way. Third, a central finding in the moral psychology literature is that differences in moral values and attitudes lead to intolerance. For example, the more morally convicted people are on a particular issue (i.e., the more their stance is grounded in their fundamental beliefs about what is right or wrong(e.g., [79])), the more they prefer to distance themselves socially from those who are attitudinally dissimilar [16, 49]. In a similar vein, we expected individuals who score high (vs. low) on the MRS to be more inclined to distance themselves socially from someone who acts in an irrational (vs. rational) way. Thus, in summary, we expected scores on the MRS to moderate moral judgments, attributions of blame, moral trait ascriptions, and social distancing from a person who violates (vs. upholds) rational principles of belief formation.

In Study 6 we tested these predictions in three different domains where reliance on beliefs that are not based on reason and evidence is widespread. Specifically, we tested the predictive validity of the MRS in the domains of astrology, homeopathy (a kind of alternative medicine), and creationism. Irrespective of domain, we expected individuals who score high (vs. low) on the MRS to be harsher in their morally laden reactions (moral judgments, character evaluations, attributions of blame, social distancing) of individuals who acted in ways that violated (vs. upheld) rational principles. Moreover, we expected these effects to emerge while statistically controlling for individual differences in the perceived (amoral) importance of rationality (IRS).

Another well-established finding in the moral psychology literature is that reactions to violations of moral rules and values are associated with negative moral emotions [80], as well as with motivation to punish the transgressor [77, 81, 82]. We therefore expected those who score high (vs. low) on the MRS to express negative moral emotions (contempt, anger, and disgust) towards someone who acts based on an irrational (vs. rational) belief. Moreover, we also expected individuals who score high (vs. low) on the MRS to express a desire for punishment of someone who acts based on an irrational belief, but not of someone who acts based on a rational belief. We tested these predictions in Study 7.

Standpoints that are rooted in morality are perceived as universally applicable “oughts”, which means that those who hold a certain moral standpoint believe that everyone should adhere to it [8, 9, 10, 11]. Moral values and attitudes are therefore particularly strong predictors of societal and political engagement, activism, and of support for groups that personify such values and attitudes [53, 54, 57, 58, 59]. In Study 8 we tested moralized rationality’s status as a universal moral “ought” more directly. Specifically, we examined whether individual differences in moralized rationality uniquely predict motivation to contribute to a charity that serves to prevent the spread of irrational beliefs in society.

## Study 6: Testing the Predictive Validity of the MRS: Moral Judgments, Moral Trait Inferences, Attributions of Blame and Social Distancing

### Method

#### Participants and design

This study was approved by the Ethics Committee of the Social, Environmental, and Organizational Psychology Research Group (S.E.O.R.G.) at University of Exeter. Six hundred US citizens were recruited from Amazon Mechanical Turk. Thirteen individuals failed to click through to the last page, resulting in a final sample of 613 participants. The study lasted approximately 15 minutes, and participants received a $1.50 payment. Fifty-seven percent were male and 43% were female (*M*_age_ = 34.90, *SD* = 11.76). In this sample 78.3% self-identified as Caucasian, 8.3% as Asian American, 6.2% as African American, 5.2% as Hispanic/Latino, 0.5% as Native American, 1.3% as other, and 0.2% did not disclose their race/ethnicity. Participants were randomly assigned to conditions in a 3 (Domain: astrology/creationism/homeopathy) x 2 (Target rationality: rational/irrational) between subjects factorial design. The MRS served as a third independent variable, and the IRS served as a control variable.

#### Procedure and materials

Upon giving their informed consent, participants filled out the MRS, IRS, and the MFQ. As in the previous studies, the items of the MRS and IRS scales were mixed together, and the order of the questions was randomized. After that, participants were randomly assigned to the astrology, creationism, or the homeopathy conditions. First, participants were asked to read a very brief and neutral description of a person called John. The intention was to enable us to assess baseline preferred social distance from this target. Preferred social distance [16] was measured using the stem “To what extent would you be happy or unhappy to have John…”, followed by (1) as your neighbor, (2) as a colleague, (3) marry into your family, (4) as a teacher to your children, and (5) as your close personal friend (1 = *very unhappy*, 7 = *very happy*). These items were then recoded such that higher scores indicated a desire for more social distance. We then averaged the items to generate a measure of baseline social distance to the target (α = .93).

The baseline social distance measure was followed by the target rationality manipulation. To manipulate target rationality, we presented some more detailed information about John (all manipulations are presented in S2 Text). In the irrational condition, John either decided to invest in a particular stock based on his belief in astrology (astrology domain), relied on an alternative medicine to treat a medical condition (homeopathy domain), or endorsed the biblical creation account of our origins (creationism domain). In the rational condition, John instead either decided to invest his money in a certain stock based on a rational analysis of highly relevant information (astrology domain), relied on regular medicine to treat a medical condition (homeopathy domain), or endorsed the theory of evolution as the explanation of our origins (creationism domain).

Upon reading one of the scenarios containing our target rationality manipulation, participants were asked to answer a set of additional questions constituting our dependent variables. First, we once again measured preferred social distance from John (post-measure), as well as moral judgments of John’s behavior. After that we measured evaluations of John’s moral character. For exploratory purposes, we also measured evaluations of John on two other central dimensions of person perception (competence and warmth; [50, 51]). In addition, we measured to what extent John was perceived as blameworthy (vs. praiseworthy). Finally, participants filled out the demographic questions, were thanked, and paid for their participation. Below we describe all the dependent variables in more detail.

Preferred social distance to the target was measured using the same items as for the measure of preferred social distance at baseline. The items were recoded such that higher scores indicated more social distance, and were then averaged to create a post-measure of preferred social distance (α = .96). Finally, we subtracted participants’ baseline social distance scores from their scores on the post-measure of preferred social distance to create a measure of *social distancing* (vs. approach). Thus, a positive score on this measure indicates distancing from the target compared to baseline, whereas a negative score indicates approach of the target compared to baseline.

Two items were used to measure *moral judgments* of John’s behavior: Specifically, we asked: “To what extent do you think what John did was moral?”, and “To what extent do you think what John did was immoral?” (1 = *not at all*, 7 = *very much*). After reversing scores on the second item, scores on the two items were averaged (*r* = .42, *p* < .001).

To measure how competent, warm and moral John was perceived to be, we presented participants with the stem: “My impression of John is that he is…” followed by a series of 7-point semantic differentials. To measure perceived competence, we used the following differentials: 1 = *very incompetent*, 7 = *very competent*; and 1 = *very irrational*, 7 = *very rational*. These two items were averaged to create a measure of *ascribed competence* (*r* = .84, *p* < .001). For warmth we used the following differentials: 1 = *very cold*, 7 = *very warm*; and 1 = *very unfriendly*, 7 = *very friendly*. These two items were averaged to create a measure of *ascribed warmth* (*r* = .85, *p* < .001). Finally, to measure perceived morality, we used three items: *very immoral*, 7 = *very moral*; 1 = *very bad*, 7 = *very good*; and 1 = *very unethical*, 7 = *very ethical*. These items were averaged to create a measure of *ascribed morality* (α = .93).

To measure attributions, we used two items: “To what extent do you think what John did was blameworthy?” and “To what extent do you think what John did was praiseworthy?” (1 = *not at all*, 7 = *very much*). Scores on the second item were reversed, after which the two items were averaged to create a measure of *attributions of blame* (*r* = .44, *p* < .001).

### Results and discussion

As in the previous studies, we filtered out participants who took part more than once (*n* = 17) as well as extremely fast responders (*n* = 17), resulting in a final sample of 579. Means, standard deviations and zero-order correlations between all of the variables are presented in Table 5. The dependent variables were analyzed with a multivariate analysis of variance (MANOVA), using domain (astrology vs. homeopathy vs. creationism), target rationality (rational vs. irrational), MRS, as well as their two-way and three-way interactions as predictors. In these analyses we also controlled for the IRS, and for its two-way and three-way interactions with domain and target rationality [83]. Where significant multivariate effects were found, we interpreted the underlying significant univariate effects using hierarchical multiple regression and simple slope analysis [84]. Due to a programming error, we lost some of the data from 40 participants in the Astrology condition. Specifically, the programming error caused the program not to present to these participants the questions used to measure moral judgments, person perception, and attributions of blame. Other variations in reported degrees of freedom are the result of listwise deletions of missing values.

**Table 5:** Means, Standard Deviations, and Zero-order Correlations (Study 6).

Variable	*M*	*SD*	1	2	3	4	5	6	7	8
1 MRS	3.70	1.25	-							
2 IRS	5.64	0.98	.33*	-						
3 Moral judgment	5.40	1.34	-.11*	.06	-					
4 Ascribed morality	5.10	1.20	.03	.09*	.59*	-				
5 Ascribed warmth	5.24	1.16	.01	.09*	.45*	.76*	-			
6 Ascribed competence	4.95	1.65	-.07	.00	.62*	.72*	.55*	-		
7 Attributions of blame (vs. praise)	3.12	1.56	.03	.01	-.73*	.57*	-.41*	-.73*	-	
8 Social distancing	0.39	1.22	.02	.06	-.56*	-.45*	-.32*	-.68*	.71*	-

* *p* < .05

#### Multivariate analyses

The results showed significant overall effects of target rationality, *F*(6, 514) = 44.79, *p* < .001, and MRS, *F*(6, 514) = 5.29, *p* < .001, which were qualified by the expected interaction between MRS and target rationality, *F*(6, 514) = 4.62, *p* < .001. Neither the MRS by domain interaction, *F*(12, 1030) = .70, *p* = .75, nor the MRS by target rationality by domain interaction, *F*(12, 1030) = 0.72, *p* = .74. Domain did however have a multivariate main effect, *F*(12, 1030) = 8.81, *p* < .001, as well as an interaction with target rationality, *F*(12, 1030) = 4.64, *p* < .001, which indicated that participants gave harsher moral judgments in the domain of creationism than in the other two domains, and that the effects of target rationality were somewhat weaker in the domain of homeopathy than in the other two domains.

Inspection of the underlying univariate effects revealed interactions between target rationality and the MRS on moral judgment, *F*(1, 519) = 18.03, *p* < .001, ascribed morality, *F*(1, 519) = 9.82, *p* = .002, ascribed warmth, *F*(1, 519) = 6.37, *p* = .01, attributions of blame/praise, *F*(1, 519) = 7.41, *p* = .007, and social distancing, *F*(1, 519) = 4.91, *p* = .03, but not on ascribed competence, *F*(1, 519) = 1.35, *p* = .25. Significant MRS by target rationality interactions were further analyzed with hierarchical multiple regression and simple slope analysis, using the MRS, target rationality and their interaction as predictors.

#### Moral judgment

**Fig 1:**
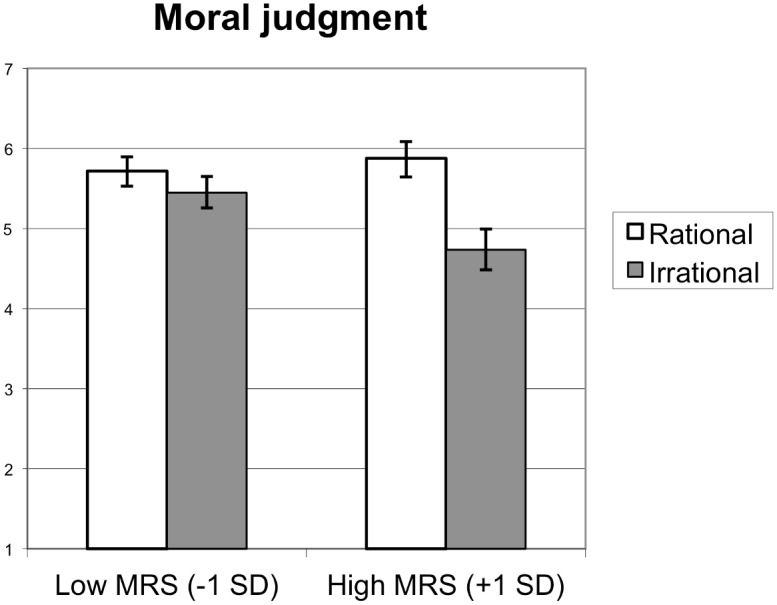
Moral Judgment. Judgment of the Target’s Actions as Moral (vs. Immoral) as a Function of Target Rationality and Moralized Rationality (Study 6). Error Bars Represent Bootstrapped (Accelerated and Bias-corrected) 95% Confidence Intervals.

As hypothesized, MRS and target rationality interacted to predict moral judgment, *B* = 0.22, *SE* = 0.05, *t*(534) = 4.15, *p* < .001, ΔR^2^ = .03 (see Fig 1). The target’s actions were seen as less moral in the irrational condition (*M* = 4.74) than in the rational condition (*M* = 5.88) among individuals high in MR (+1*SD*), *t*(534) = 7.56, *p* < .001, but not among individuals low in MR (-1*SD*), *M*_irrational_ = 5.45, *M*_rational_ = 5.72, *t*(534) = 1.72, *p* = .09, an effect that replicated across all three domains studied.

#### Attributions of blame/praise

**Fig 2:**
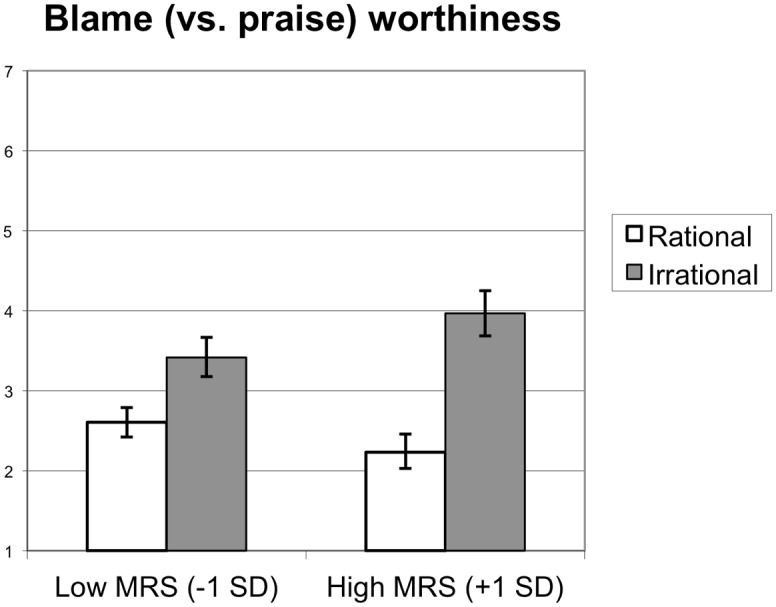
Blameworthiness. Perceived Blame (vs. Praise) Worthiness of the Target’s Actions as a Function of Target Rationality and Moralized Rationality (Study 6). Error Bars Represent Bootstrapped (Accelerated and Bias-corrected) 95% Confidence Intervals.

MRS and target rationality also interacted to predict the extent to which participants viewed the target’s actions as blameworthy (/ unpraiseworthy), *B* = -0.23, *SE* = 0.06, *t*(535) = -3.75, *p* < .001, ΔR^2^ = .02 (see Fig 2). The target’s actions were seen as more blameworthy (/ less praiseworthy) in the irrational condition (*M* = 3.97) than in the rational condition (*M* = 2.23) among individuals high in MR (+1*SD*), *t*(535) = -10.02, *p* < .001. This difference was still significant, though less pronounced, among individuals low in MR (-1*SD*), *M*_irrational_ = 3.41, *M*_rational_ = 2.60, *t*(535) = -4.73, *p* < .001.

#### Ascribed morality and warmth

**Fig 3:**
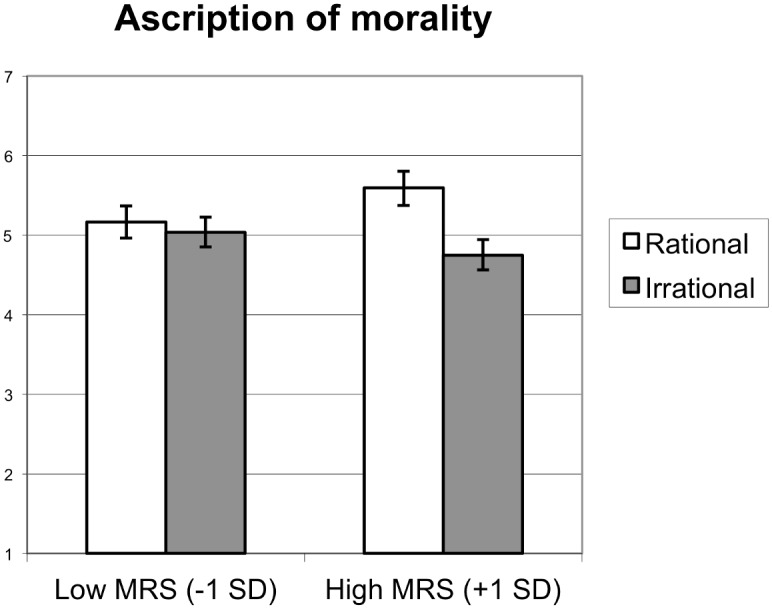
Ascribed Morality. Morality Ascribed to the Target as a Function of Target Rationality and Moralized Rationality (Study 6). Error Bars Represent Bootstrapped (Accelerated and Bias-corrected) 95% Confidence Intervals.

As hypothesized, MRS and target rationality interacted to predict to what extent the target was seen as moral, *B* = 0.18, *SE* = 0.05, *t*(536) = 3.61, *p* < .001, ΔR^2^ = .02 (Fig 3), and warm, *B* = 0.10, *SE* = 0.05, *t*(535) = 2.10, *p* = .04, ΔR^2^ = .01. The interaction between MRS and target rationality on the ascription of warmth disappeared when we controlled for ascriptions of morality (*p* = .35), whereas the interaction on ascriptions of morality remained significant when we controlled for the ascription of warmth (*p* = .002). Because MRS and target rationality did not affect the ascription of warmth beyond their effects on the ascription of morality we chose not to further interpret the interaction on warmth. As predicted, the target was seen as less moral in the irrational condition (*M* = 4.75) than in the rational condition (*M* = 5.60) among individuals high in MR (+1*SD*), *t*(536) = 5.97, *p* < .001, but not among individuals low in MR (-1*SD*), *M*_irrational_ = 5.04, *M*_rational_ = 5.16, *t*(536) = 0.88, *p* = .38.

#### Social distancing

**Fig 4:**
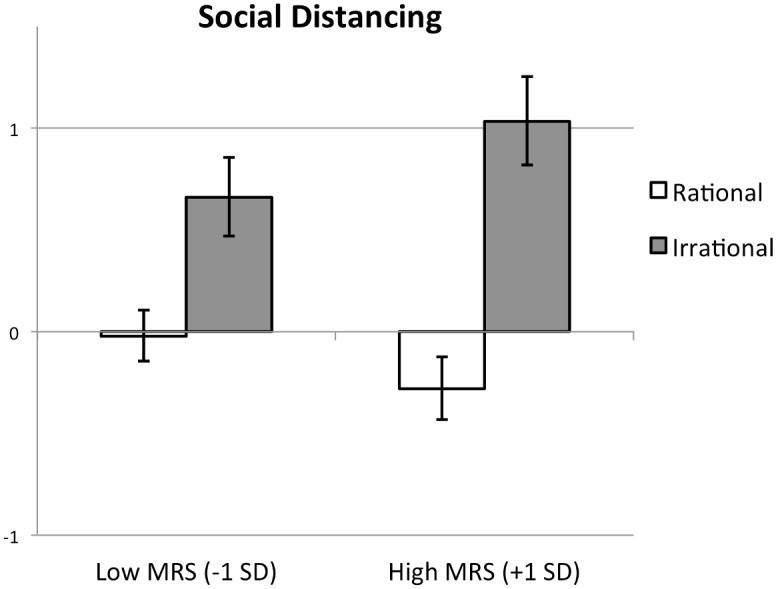
Social Distancing. Social Distancing (vs. Social Approach) as a Function of Target Rationality and Moralized Rationality (Study 6). Error Bars Represent Bootstrapped (Accelerated and Bias-corrected) 95% Confidence Intervals.

As predicted, MRS and target rationality interacted to affect participants’ preferred social distance from the target (pretest—posttest), *B* = -0.15, *SE* = 0.05, *t*(567) = -3.36, *p* = .001, ΔR^2^ = .02, Fig 4. Among individuals high in MR (+1*SD*) there was a strong distancing response to the irrational target (*M* = 1.03), and a weaker approach response to the rational target (*M* = -0.28), *t*(567) = -10.10, *p* < .001. This difference was still significant, though less pronounced, among individuals low in MR, *M*_irrational_ = 0.66, *M*_rational_ = -0.02, *t*(567) = -5.38, *p* < .001.

We also tested the scores on the distancing measure against 0, a value that indicates no movement in individuals’ preferred social distance to the target compared to baseline. The results showed that among individuals high in MR (+1 *SD*) the target elicited a significant social distancing response in the irrational condition (*M* = 1.05, *t*[283] = 9.65, *p* < .001), and a significant social approach response in the rational condition (*M* = -0.28, *t*[271] = -3.78, *p* < .001). Among individuals low in MR (-1 *SD*), the target elicited a significant social distancing response in the irrational condition (*M* = 0.71, *t*[283] = 6.40, *p* < .001), and a non-significant distancing response in the rational condition (*M* = 0.08, *t*[271] = 0.11, *p* = .91).

Some researchers have argued against the use of difference scores in the analysis of pre-test post-test designs, and instead recommend controlling for the pre-test in the analysis of the post-test [85]. To rule out that the present findings resulted from idiosyncrasies of the difference score we used, we re-analyzed the post-measure of preferred social distance to the target while controlling for the pre-measure. This alternative analysis yielded virtually identical results. Specifically, the predicted interaction between moralized rationality and target rationality was obtained, *B* = -0.17, *SE* = 0.05, *t*(553) = -3.53, *p* < .001. As expected, individuals high in MR (+ 1 SD) desired greater social distance from the irrational target than from the rational target, *B* = -0.67, *SE* = 0.07, *t*(553) = -10.18, *p* < .001. This difference was still significant, though much less pronounced, among individuals low in MR (- 1 SD), *B* = -0.34, *SE* = 0.07, *t*(553) = -5.23, *p* < .001.

We used bootstrapped mediated moderation analysis [86]to investigate the process responsible for the finding that MR increased the effect of target rationality on social distancing (Model 7, 5000 resamples, bias corrected). We expected that individuals high (vs. low) in MR would desire greater distance to an irrational other than to a rational other because they should see irrational others as less moral. We thus tested whether the perceived morality of the target (i.e., ascribed morality) explains why social distancing from the irrational (vs. rational) target was greater among individuals high (vs. low) in MR. The results showed support for this prediction; index of mediated moderation: -0.07, *SE* = 0.02, 95% *CI*: (-0.11, -0.03). As expected, we found an indirect effect of target rationality on distancing responses to the target through the target’s perceived morality among individuals high in MR (+1 *SD*), estimate: -0.16, *SE* = 0.03, 95% *CI*: (-0.24, -0.10), but not among individuals low in MR (-1*SD*), estimate: -0.02, *SE* = 0.03, 95% *CI*: (-0.08, 0.03).

The results of Study 6 thus show that viewing a reliance on rational means of forming and evaluating beliefs as a moral issue affects how people respond to the behavior of others.

Across three different domains (homeopathy, astrology, creationism), individuals high in MR responded more negatively to irrational behavior than individuals low in MR. More specifically, individuals high (vs. low) in MR judged irrational behavior as more immoral than rational behavior, and perceived others engaging in rational behavior as more moral than individuals engaging in irrational behavior. Furthermore, individuals high in MR judged irrational behavior to be more blameworthy, and distanced themselves more from an irrational target person than individuals low in MR. As expected, these effects were independent of individuals’ perceived personal importance of rationality. Thus, the results of Study 6 show that MR not only alters moral judgments of others’ beliefs and behavior as a function of their rationality; it also shows that these moral judgments have important downstream consequences for interpersonal intolerance.

To conceptually replicate and extend this initial evidence of the consequences of moral rationality, in Study 7 we examined the implications of moralized rationality for two different indicators of interpersonal intolerance. Specifically, we investigated whether those who moralize rationality are uniquely inclined to express (1) negative moral emotions towards, and (2) a desire for punishment of someone who acts based on an irrational (vs. rational) belief.

## Study 7: Testing the Predictive Validity of the MRS: Moral Emotions and Punishment

### Method

#### Participants and design

This study was approved by the Office for the Protection of Research Subjects (OPRS) at the University of Illinois at Chicago (Protocol 2015–1017). Two hundred and sixty US citizens were recruited from Amazon Mechanical Turk. Two individuals failed to click through to the very last page, which resulted in a final sample of 262 participants. The study lasted approximately 10 minutes, and participants received $1 as remuneration. Fifty-two percent were male and 48% were female (*M*_age_ = 33.61, *SD* = 10.01). In this sample 80.2% self-identified as Caucasian, 7.5% as Asian American, 5.9% as African American, 5.1% as Hispanic/Latino, 0.4% as Native American, and 0.8% as other. Participants were randomly assigned to the conditions of a one factor (Target rationality: rational/irrational) between subjects factorial design. The MRS served as a second independent variable, and the IRS served as a control variable.

#### Procedure and materials

After giving their informed consent, participants filled out the MRS, IRS, and the MFQ. As in the previous studies, the items of the MRS and IRS scales were mixed together, and the order of the questions was randomized. Participants were then asked to read a short scenario, which described an interaction between a doctor (Richard, who served as the target in this study) and his patient (Mary). As described in the scenario, Richard advised the devoutly Christian Mary (who suffered from unexplained diffuse symptoms) to pray to God for better health. Richard’s reason for advising Mary to pray was varied experimentally to manipulate the rationality of his belief that prayer would help Mary. In the rational target condition, Richard advised Mary to pray for better health because he believed prayer would function as a placebo for Mary. By contrast, in the irrational target condition, Richard advised Mary to pray for better health because he believed God answers the prayers of the faithful (the text used to manipulate target rationality in this study is presented in S3 Text). Upon reading one of the scenarios containing our target rationality manipulation, participants were asked to answer a set of questions, which constituted the dependent variables. Finally, participants answered some demographics questions and were thanked for their participation.

*Moral emotions* towards the target [80] were measured with 3 items (α = .94). Participants indicated on 7-point rating scales (1 = *not at all*, 7 = *very much*) the extent to which thinking about Richard’s actions made them feel angry at Richard / disgusted by Richard / contempt for Richard.

*Desire for punishment* of the target was measured with 6 items (α = .96). Specifically, we asked: “Does Richard deserve to be punished for his behavior?”, “Should Richard be punished for his actions”, “Do you wish that Richard would be punished?” (1 = *not* at all, 7 = *very much*), “Richard doesn’t deserve to be punished for his behavior.”, “Richard’s actions don’t warrant any punishment.”, and “Richard’s actions should not be permissible and deserve punishment.” (1 = *completely disagree*, 7 = *completely agree*). The fourth and fifth items of the scale were reverse-scored. Means, standard deviations, and correlations of the variables that were measured in Study 7 are presented in Table 6.

**Table 6:** Means, Standard Deviations and Zero-order Correlations (Study 7).

Variable	*M*	*SD*	1	2	3	4
1 MRS	3.78	1.27	-			
2 IRS	5.68	0.91	.34*	-		
3 Moral emotions	2.94	1.99	.26*	.05	-	
4 Punishment	2.61	1.82	.24*	-.03	.81*	-

* *p* < .05

### Results and discussion

As in previous studies, we filtered out extremely fast responders (*n* = 9), resulting in a final sample of 253. The dependent variables were analyzed with a multivariate analysis of variance (MANOVA), using target rationality (rational vs. irrational), MRS, and their two-way interaction as predictors. In this analysis we controlled for the IRS, and for its two-way interaction with target rationality [83]. Where significant multivariate interaction effects were found, we interpreted the underlying significant univariate effects using hierarchical multiple regression and simple slope analysis [84].

#### Multivariate analyses

The results showed significant overall effects of target rationality, *F*(2, 238) = 12.79, *p* < .001, and MRS, *F*(2, 238) = 12.57, *p* < .001, which were qualified by the expected interaction between MRS and target rationality, *F*(2, 238) = 3.83, *p* = .02.

#### Moral emotions

**Fig 5:**
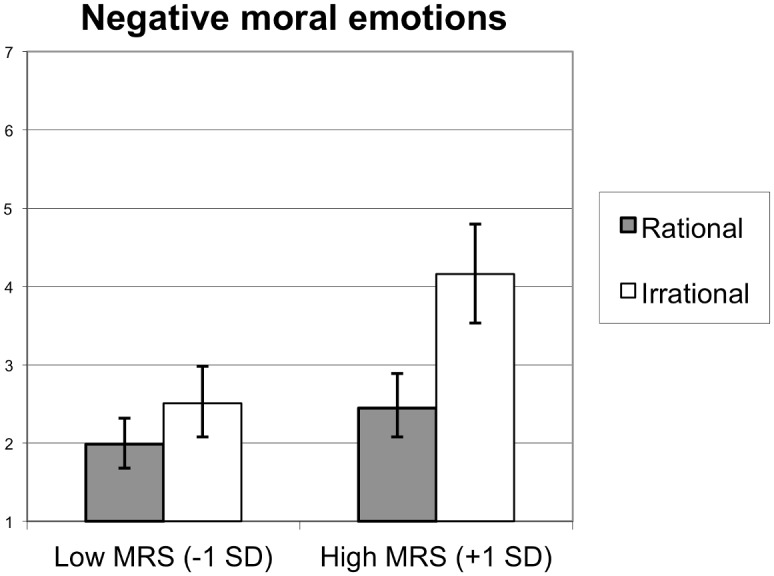
Moral Emotions. Negative Moral Emotions Towards the Target as a Function of Target Rationality and Moralized Rationality (Study 7). Error Bars Represent Bootstrapped (Accelerated and Bias-corrected) 95% Confidence Intervals.

As hypothesized, MRS and target rationality interacted to predict the extent to which participants experience negative moral emotions towards the target, *B* = 0.30, *SE* = 0.12, *t*(244) = 2.57, *p* = .01, ΔR^2^ = .02 (see Fig 5). The target elicited more negative moral emotions in the irrational condition (*M* = 4.16) than in the rational condition (*M* = 2.45) among individuals high in MR (+1*SD*), *t*(244) = 5.22, *p* < .001. No such difference was found among individuals low in MR (-1*SD*), *M*_irrational_ = 2.51, *M*_rational_ = 1.99, *t*(244) = 1.62, *p* = .11.

#### Desire for punishment

**Fig 6:**
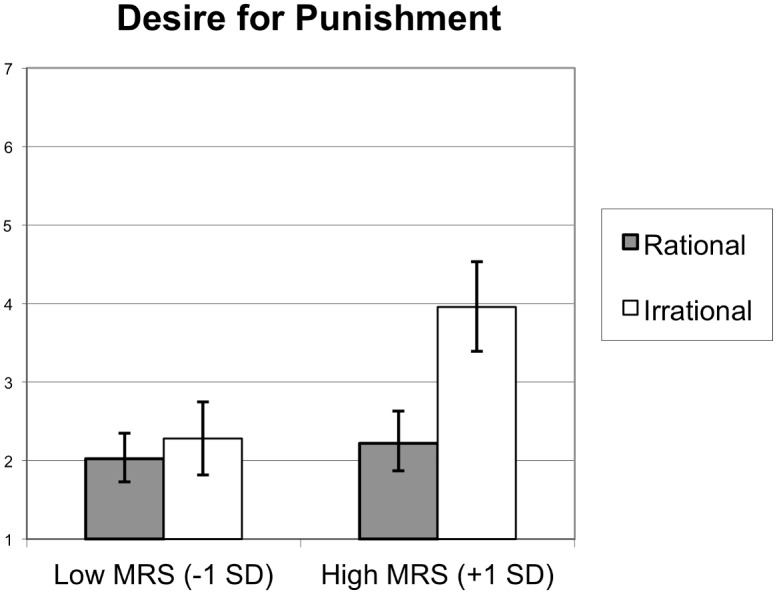
Punitive Desires. Desire for Punishment of the Target as a Function of Target Rationality and Moralized Rationality (Study 7). Error Bars Represent Bootstrapped (Accelerated and Bias-corrected) 95% Confidence Intervals.

As hypothesized, MRS and target rationality interacted to predict the extent to which participants desired that the target be punished for his behavior, *B* = 0.37, *SE* = 0.11, *t*(243) = 3.44, *p* < .001, ΔR^2^ = .04 (see Fig 6). Participants high in MR (+1*SD*) desired the irrational target to be punished more severely (*M* = 3.96) than the rational target (*M* = 2.22), *t*(243) = 5.72, *p* < .001. No such difference was found among individuals low in MR (-1*SD*), *M*_irrational_ = 2.28, *M*_rational_ = 2.02, *t*(243) = 0.86, *p* = .39. Additional analyses revealed that the interaction between the MRS and the manipulation of the target’s stance remained significant, and in the predicted direction, (*B* = 0.29, *SE* = 0.11, *t*(237) = 2.67, *p* = .008) when we controlled for religiosity [69], and for its interaction with target’s stance. Similarly, the interaction between the MRS and target’s stance remained highly significant, and in the predicted direction (*B* = 0.38, *SE* = 0.11, *t*(237) = 3.45, *p* < .001), when we controlled for the moral foundation of Care/harm [53], as well as for its interaction with target’s stance. Individual differences in religiosity or in the endorsement of Care/harm as a moral value therefore do not explain why individuals high in MR desired more punishment for the irrational target than for the rational target.

We used bootstrapped mediated moderation analysis [86] to investigate whether the experience of negative moral emotions could explain why the desire for punishment of the target was higher in the irrational target condition than in the rational target condition among individuals high (vs. low) in MR (Model 7, 5000 resamples, bias corrected). The results showed support for this idea, index of mediated moderation: 0.34, *SE* = 0.15, 95% *CI*: (0.04, 0.63). As expected, we found a strong indirect effect of target irrationality (vs. rationality), through negative moral emotion, on desire for punishment among people high in MR (+1*SD*), estimate: 1.25, *SE* = 0.30, 95% *CI*: (0.65, 1.81), but not among people low in MR (-1*SD*), estimate: 0.40, *SE* = 0.21, 95% *CI*: (-0.01, 0.81).

Studies 6 and 7 have demonstrated that individual differences in moralized rationality have important downstream consequences for various indicators of intolerance of irrational beliefs, acts, and actors. In the final study we wanted to examine the effects of the MRS on another important phenomenon typically associated with strong moral values and attitudes -- motivation to engage in activism.

## Study 8: Testing the Predictive Validity of the MRS: Motivation to Prevent the Spread of Irrational Beliefs

A unique feature of moral (vs. amoral) values and attitudes is that they are perceived as “oughts” rather than as preferences, as objectively true, and as universally applicable to everyone, everywhere [8, 9, 10, 11]. It is therefore not surprising that morally grounded positions are strong predictors of societal and political engagement and activism in support of those positions, and of support for groups that personify such values [53, 54, 55, 56, 57, 58, 59]. In Study 8 we therefore wished to investigate whether endorsement of rationality as a moral value would be associated with an increased willingness to contribute to a charity that aims to prevent the spread of irrational beliefs. To test this idea, participants in Study 8 were introduced to six (fictitious) charities that were each specifically constructed to serve one of several different moral values. Five of the charity descriptions were designed to map onto five of the moral foundations (i.e., Care/harm, Fairness/cheating, Loyalty/betrayal, Authority/subversion and Sanctity/degradation). Critically, the sixth charity was designed to map onto rationality, as its aims were to prevent the spread of irrational beliefs in society. Participants’ willingness to volunteer for, and to donate money to each of the six charities served as the dependent variables. We hypothesized that the MRS should be a unique predictor of people’s willingness to contribute to the charity that served to prevent the spread of irrational beliefs in society, but not to any of the other charities.

Finally, a potential limitation of Studies 1 through 7 is that they relied exclusively on the use of the MTurk panel as a source of participants. In Study 8 we wished to address this potential limitation, and investigate the predictive utility of the MRS outside of this population. Participants for Study 8 were therefore recruited from a University with a demographically diverse population of students, faculty, and staff.

### Method

#### Sample

This study was approved by the Office for the Protection of Research Subjects (OPRS) at the University of Illinois at Chicago (Protocol 2015–1017). Three hundred and eleven students, faculty and staff at the University of Illinois at Chicago were recruited through this university’s mass mail system to take part in a fifteen-minute online study. In exchange for their participation, they were offered a chance to win a $50 gift certificate in a raffle. Thirty-three percent of respondents were male and 67% were female (*M*_age_ = 31.56, *SD* = 13.78). Fifty-one percent self-identified as Caucasian, 18% as Asian American, 16% as Hispanic/Latino, 8% as African American, 1% as Native American, and 6% as other. The level of education in the sample was as follows: 0.3% reported having less than a high school degree, 4.5% had a high school degree, 30.5% had some college education (but no diploma), 28.6% had a Bachelor degree, 23.2% had a Master’s degree, 8.7% had a Doctoral degree, and 4.2% opted not to report their level of education.

#### Procedure and materials

Upon giving their informed consent, participants filled out the MRS, IRS, and the MFQ. As in the previous studies, the items of the MRS and IRS were mixed together, and the order of the questions was randomized. Participants were then asked to read the descriptions of six different charities, five of which were tailored to embody the moral foundations of Care/harm (*Project Compassion*), Fairness/cheating (*Justice for All*), Loyalty/betrayal (*Giving Back to our Heroes*), Authority/subversion (*In the Line of Duty*), and Sanctity/degradation (*Worth the Wait*). Crucially, the sixth charity (*Skeptic Alliance*) was specifically designed to reflect a societal commitment to rationality, as its objective was to prevent the spread of irrational beliefs in society. The order in which the six charities were presented was randomized. The descriptions of all six charities are presented in S4 Text.

*Participants’ willingness to contribute to each charity* was assessed with two items per charity, administered directly following the introduction of that charity. First, participants were asked to which extent they would be willing to volunteer for the charity (e.g., “Would you be willing to volunteer for the cause of *Skeptic Alliance*?” [1 = *not at all*, 7 = *very much*]). Second, they were asked to which extent they would be willing to donate money to the charity (e.g., “Would you be willing to donate money to *Skeptic Alliance*?” [1 = *not at all*, 7 = *very much*]).

As an additional and more conservative test of our hypothesis, we asked participants how they would prefer to divide a limited amount of money ($50) between the six charities. We expected that, even when in zero-sum competition with the other charities, the MRS would be positively related to individuals’ inclination to donate money to the rational charity. Finally, after filling out some demographic questions, participants were thanked for their participation.

### Results and discussion

Means, standard deviations and zero-order correlations between all of the variables are presented in Table 7. The dependent variables were analyzed with regression analysis, using the MRS, IRS, and endorsement of each of the five moral foundations as predictors.

**Table 7:** Means, Standard Deviations, and Zero-order Correlations (Study 8).

Variable	*M*	*SD*	1	2	3	4	5	6	7	8	9	10
1 MRS	3.76	1.01	-									
2 IRS	5.61	0.90	.42*	-								
3 Willing to donate to Skeptic Alliance	2.85	1.91	.33*	.29*	-							
4 Willing to volunteer for Skeptic Alliance	3.14	2.07	.34*	.29*	.83*	-						
5 Donation to Skeptic Alliance	4.18	7.58	.35*	.25*	.44*	.42*	-					
6 Donation to Project Compassion	10.71	11.41	-.04	-.12*	-.10	-.10	-.15*	-				
7 Donation to Justice for All	15.89	13.54	-.01	.06	-.05	-.05	-.23*	-.41*	-			
8 Donation to In the Line of Duty	6.81	6.37	-.11	-.04	.05	.04	-.08	-.30*	-.36*	-		
9 Donation to Giving Back to our Heroes	9.37	8.08	-.12*	-.03	-.11*	-.12*	-.20*	-.23*	-.40*	.26*	-	
10 Donation to Worth the Wait	3.01	6.10	-.04	-.11*	-.10	-.10	-.10	-.16*	-.27*	.06	-.03	-

* *p* < .05

#### Willingness to volunteer

As predicted, the extent to which participants moralized rationality (MRS) was positively related to their willingness to volunteer for the rationality-oriented charity (Skeptic Alliance), *b* = 0.54, *SE* = 0.13, *t*(281) = 4.02, *p* < .001, but not for any of the other charities (*p*’s > .24). The personal importance participants attached to rationality (IRS) had only a marginally significant effect on willingness to volunteer, *b* = 0.27, *SE* = 0.15, *t*(281) = 1.86, *p* = .06. Out of all five moral foundations, only endorsement of Sanctity/degradation as a moral value was significantly (negatively) related to participants’ willingness to volunteer for Skeptic Alliance, *b* = -0.53, *SE* = 0.15, *t*(281) = -3.53, *p* < .001. None of the other moral foundations were significantly related to individuals’ willingness to volunteer for the Skeptic Alliance, *p*’s > .21. Thus, endorsement of rationality as a moral value was uniquely associated with individuals’ willingness to volunteer for the rationality-oriented charity.

#### Willingness to donate

Also as predicted, the MRS was positively related to individuals’ willingness to donate money to the rationality-oriented charity, *b* = 0.48, *SE* = 0.12, *t*(280) = 4.17, *p* < .001, but not to any of the other charities (*p*’s > .26). In addition, the IRS again had a marginally significant effect on the willingness to donate to the rationality-oriented charity, *b* = 0.26, *SE* = 0.13, *t*(280) = 1.97, *p* = .05. Furthermore, endorsement of Sanctity/degradation as a moral value was significantly (negatively) related to participants’ willingness to donate to the rationalist charity, *b* = -0.34, *SE* = 0.14, *t*(280) = -2.46, *p* = .01. None of the other moral foundations were significantly related to individuals’ willingness to donate to the rationalist charity, *p*’s > 16. Thus, endorsement of rationality as a moral good was uniquely associated with individuals’ willingness to donate to the rationality-oriented charity.

#### Donations

As an additional test of our hypothesis, we asked participants to divide $50 between the six charities. As predicted, even under such zero-sum conditions, the endorsement of rationality as a moral value was positively associated with donations to the rationality-oriented Skeptic Alliance, *b* = 2.02, *SE* = 0.43, *t*(280) = 4.68, *p* < .001, but not to donations to any of the other charities, *p*’s > .06. Donations to the rationality-oriented charity were only marginally related to the IRS, *b* = 0.95, *SE* = 0.50, *t*(280) = 1.88, *p* = .06. Notably, both Care/harm, *b* = -2.48, *SE* = 0.74, *t*(280) = -3.34, *p* = .001, and Sanctity/degradation, *b* = -1.23, *SE* = 0.52, *t*(280) = 2.38, *p* = .02, were negatively related to donations to the rationality-oriented Skeptic Alliance. However, given the zero-sum nature of the donation measure, the strong effects of Care/harm (*b* = 3.82, *SE* = 1.27, *t*(280) = 3.01, *p* = .003) and Sanctity/degradation (*b* = 2.80, *SE* = 0.43, *t*(280) = 6.48, *p* < .001) on donations to their respective charities may explain these findings.

Moral values and attitudes are perceived as “oughts” rather than as personal preferences, as objectively true, and as universally applicable. They therefore serve as strong motivators of societal engagement, and of support for value-relevant groups [53, 54, 55, 56, 57, 58, 59]. We therefore expected that the endorsement of rationality as a moral value would be related to individuals’ willingness to support a charity that aimed to promote rational inquiry and to prevent the spread of irrational belief in society. The results of Study 8 showed support for this prediction; the MRS was strongly positively related to the willingness to volunteer for, and donate to, the rationality-oriented Skeptic Alliance, even when it was placed in zero-sum competition with other charities over limited resources. Furthermore, the results of Study 8 showed that endorsement of rationality as a moral value was unrelated to willingness to contribute to any of the other charities, a finding that further attests to the specificity of the MRS. The finding that the MRS was uniquely associated with motivation to contribute to a societal group that aimed to prevent the spread of irrational beliefs represents further evidence that the MRS taps into a moral construct [53].

Study 8 extends the results of studies 6 and 7 in several important ways. First of all, by sampling from a different population, Study 8 provides evidence for the generalizability of the utility the MRS has for understanding social behavior. Furthermore, by focusing on societal engagement as a dependent variable, Study 8 demonstrates that moralized rationality has consequences beyond interpersonal evaluations and behavior. Indeed, it suggests that support for real-world groups such as *The Skeptics Society* is likely to stem from the moralization of a reliance on reason and evidence in the formation and evaluation of belief.

## General Discussion

In this article we set out to investigate whether some people view it as a virtue to rely on reason and evidence when forming and evaluating beliefs, and whether such a moral stance has any interpersonal consequences. Eight studies provided empirical support for the notion that there are meaningful stable individual differences in the extent to which people moralize a reliance on reason and evidence when forming and evaluating beliefs. Studies 1–3 demonstrated that the scale we developed to measure individual differences in moralized rationality (MRS) is internally consistent, as well as that it measures something conceptually distinct from the personal importance attached to being rational [16].

**Fig 7:**
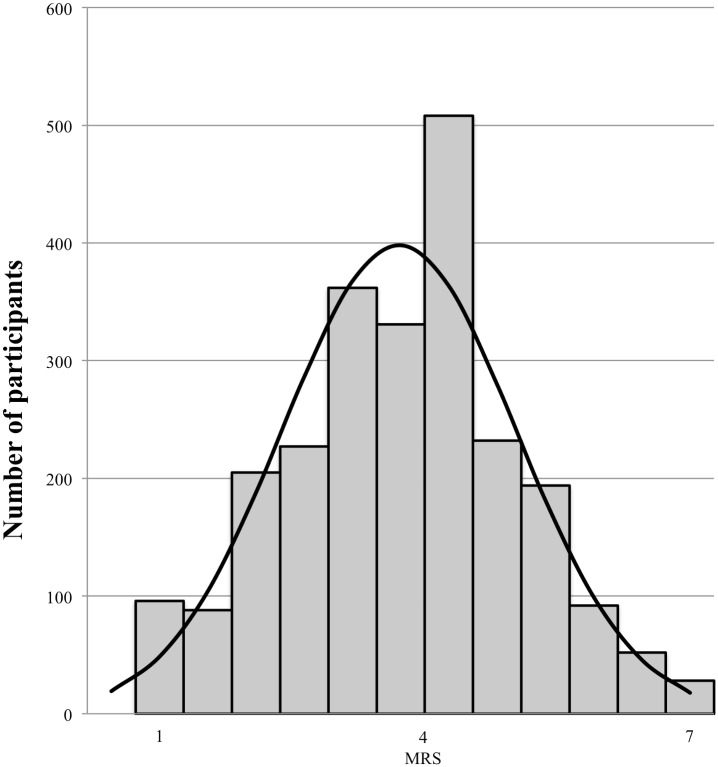
Moralized Rationality Scores. The Distribution of Scores on the MRS in Half Scale-point Intervals Compared to a Normal Distribution with the Same Mean and Standard Deviation (Combined Data from Studies 1, 2, 3, 5, 6, 7, and 8).

Study 4 demonstrated that scores on the MRS are highly stable over time, and that they were equally stable over a four-month time interval as over a two-month time interval. In addition, as can be seen in Fig 7, MRS scores spanned the whole width of the scale and were fairly normally distributed. Study 5 provided support for the convergent and discriminant validity of the MRS. As expected, the inclination to moralize rationality was positively associated with a belief in science, and negatively related to paranormal beliefs and religiosity. Individuals who moralized rationality were also uniquely inclined to view rationality-oriented traits as prototypical of a moral role model, and to view irrationality-oriented traits as prototypical of an immoral person. By contrast, the MRS did not uniquely predict the perceived prototypicality of other traits more typically associated with moral role models or immoral people [70], attesting to its specificity. Furthermore, the MRS was unrelated to utilitarian inclinations in moral dilemmas, and negatively related to deontological inclinations. Finally, results from five independent samples (Study 1, Study 3, Study 6, Study 7, and Study 8) strongly suggest that the inclination to moralize rationality cannot be reduced to any of the moral foundations [53, 63]. The MRS thus appears to exclusively assess the perceived moral relevance of rationality, and does not reduce to any common conceptualization of morality [53, 60, 70].

In Studies 6, 7, and 8, we demonstrated the predictive utility of the moralized rationality scale. Importantly, all of the effects of moralized rationality that were documented in these three studies were obtained while controlling for the personal importance attached to being rational. Thus, it was the unique moral component of the MRS that generated the effects. Across three different domains (astrology, alternative medicine, creationism), the MRS was found to affect morally laden reactions to acts that violated (vs. upheld) rational principles of belief formation and evaluation (Study 6). In all three domains, individuals who scored high on the MRS judged irrational (vs. rational) acts as less moral, and more blameworthy than individuals who scored low on the MRS. Similar effects were found on measures of person perception across all three domains. Individuals who scored high (vs. low) on the MRS perceived the target who acted in an irrational (vs. rational) manner as a less moral person. Consistent with previous work in moral psychology, individuals who scored high (vs. low) on the MRS were also more inclined to distance themselves socially from a target who acted in an irrational (vs. rational) way [16, 49]. Mediated moderation analyses indicated that the inclination to distance oneself from the irrational (vs. rational) target among individuals who scored high (vs. low) on the MRS was mediated by evaluations of the target as an immoral person. Results of Study 7 showed that those who moralize rationality expressed negative moral emotions towards a person who acted based on an irrational belief. Individuals who moralize rationality were also uniquely inclined to think that the person who acted based on an irrational belief deserved to be punished for it -- an effect that was mediated by negative moral emotions. Taken together, these findings demonstrate that stable individual differences in the inclination to moralize rationality uniquely explain morally laden reactions to violations of rational principles of forming and evaluating beliefs.

Finally, the results of Study 8 showed that the MRS has predictive utility with regard to another phenomenon commonly associated with morally laden values and attitudes -- motivation to engage in activism. The MRS was uniquely associated with motivation to volunteer for, and donate money to, a charity that works to prevent the spread of irrational beliefs. In fact, the MRS predicted how much money (out of $50) individuals preferred to allocate to this charity, while in zero-sum competition with five other charities that promoted causes consistent with five of the moral foundations. This latter finding is important, as it suggests that preventing the spread of irrational beliefs in society is regarded as an issue of high priority among those who score high on moralized rationality.

These findings may have important implications for our understanding of conflicts revolving around the validity of specific beliefs, such as the longstanding discussion about the teaching of creationism in science classes, as well as clashes between traditional beliefs and science more generally. The present results suggest that it is not only defenders of traditional beliefs that are spurred on by their moral conviction, but that the motives fueling advocates of science may be moral in nature as well. More specifically, these results suggest that they may be motivated by their conviction that it is morally wrong to rely on beliefs that are not backed up by logic and evidence. To the extent that this is the case, it could also help explain why their argumentative style frequently comes off as angry and intolerant. Numerous studies, including Studies 6 and 7 in the present article, have found a strong link between moral motivation and indicators of intolerance [12, 16, 49].

The present findings may also help account for the existence of various secular activist groups (e.g., *American Atheists*, *Center for Inquiry*, *Secular Coalition for America*, *The Skeptics Society*). As illustrated by the results of Study 8, moral motivation is a strong predictor of various forms of activism [54, 57, 58, 59]. Because the MRS uniquely predicted self-reported willingness to contribute to a charity that worked to prevent the spread of irrational beliefs, it seems plausible that individuals who join a secular activist group frequently do so for moral reasons, and that moralized rationality is at the core of their moral motivation.

It is important to note that although we expect the MRS to be a strong predictor of actual engagement in secular activism, we do not expect it to be strongly related to atheism per se. Just as any other (un)belief, atheism has various cultural, motivational, and cognitive antecedents [87], and we do not expect moralized rationality to play a role in all possible paths to atheism. For example, growing up in a highly secular community, in which religious displays are rare, should promote atheism [87]. However, we have no reason to believe that such an upbringing in itself should be associated with higher scores on the MRS. By contrast, we do suspect that the MRS is related to becoming an atheist through a deliberate, rational process [87]. Consistent with this analysis, we found only a small difference in moralized rationality between self-proclaimed atheists and people who reported being religiously affiliated (Christians, Muslims, Jews, or Other) in the present studies. Collapsing across all studies, atheists reported somewhat stronger endorsement of moralized rationality (*M* = 4.10, *SD* = 1.39) than the religiously affiliated (*M* = 3.58, *SD* = 1.08), *t*(1858) = 8.46, *p* < .001, Cohen’s *d* = .42. In future studies it would be interesting to examine whether the strength of the relationship between moralized rationality and atheism depends on the extent to which the path to atheism was based on deliberate reasoning.

The present work may also have important implications for research on reasoning and attitude change. Motivation to reach particular conclusions frequently influences the way people evaluate ideas and their evidence [34], and there is support for the notion that core ideological and moral values play a key role in generating such motivated reasoning biases [5]. Presumably this occurs because such core values are of particular importance to people. Whenever one’s core values are at stake, the motivation to reach a particular conclusion should therefore be ramped up considerably. Moralized rationality is different from other moral values, however, in that what is being moralized is not a particular outcome or belief, but the very process of rationally evaluating belief. It is therefore possible that individuals who moralize rationality are less susceptible to motivated reasoning biases. We are currently in the process of testing this idea empirically.

More generally, it seems plausible that individual differences in moralized rationality affect individuals’ openness to social influence and attitude change [88, 89]. For individuals high in moralized rationality, the moralization of specific beliefs depends on the rationality of the method that was used in the formation of that belief. This suggests that those who moralize rationality should be open to change their attitudes and beliefs, even those they moralize, when reason and evidence point them in another direction. Thus, whereas it is generally very difficult to change the mind of someone who has taken a moral stand on a specific issue [90, 91], individuals high in moralized rationality should be willing to change their attitudes and beliefs, even the moralized ones, when logic and evidence indicate they have been wrong. Examining how individual differences in moralized rationality affect attitude change, the processes through which attitudes change, and the susceptibility to engage in motivated reasoning, all strike us as promising avenues for future research.

Another fascinating question is whether individuals who moralize rationality are more open to consider and discuss societal issues that are generally regarded as taboo [82]. For example, some researchers have argued that parenting may have little to no effect on the personality of one’s offspring [92]—a proposition that was met with moral outrage [93]. The theoretical argument likely caused such indignation because it was perceived as inconsistent with society’s strong commitment to preventing maltreatment of children, as well as with the popular view of the human mind as a blank slate, shaped in its entirety by experience [93]. Another highly inflammatory topic is the possibility of stable group differences in cognition or behavior as a function of race or gender. This possibility is presumably so inflammatory because it seems inconsistent with our commitment to racial and gender equality [82], and because of our long history of racial and gender discrimination. We suspect that individuals who moralize rationality are less likely to condemn someone for bringing up highly inflammatory topics for discussion. They may instead respond with moral outrage when someone gets chastised for bringing up a “forbidden” topic for discussion, because they may perceive it as obstructing the rational and intellectually honest evaluation of ideas.

### Limitations

An important question left unanswered by the present research concerns the role of opportunity and capacity in relation to how beliefs are formed and evaluated. Specifically, the results of the present work are silent on the question of whether moralizing rationality always leads to condemnation of irrationality, or if it only does so under circumstances where individuals have the opportunity and capacity to make decisions based on a rational process. We suspect that the latter might be the case. When the information required to form an educated opinion on an issue is unavailable, we would not expect moralized rationality to generate the same amount of moral condemnation towards someone relying on a less rational process of forming an opinion (e.g., intuition, authority). To illustrate, despite the potential harm done, we do not think that individuals who moralize rationality would perceive Western European parents who lived during the Middle Ages as particularly immoral if they brought their child to an exorcist to address a psychological illness that was unknown at the time. When relevant information is readily available, however, those who moralize rationality should consider reliance on less rational means as immoral, blameworthy, and worthy of punishment. Thus, any Western European parents bringing their mentally ill child to an exorcist today, rather than to a doctor, should be perceived as highly immoral by those who moralize rationality. In a similar vein, the presumed cognitive capacity of the target person is also likely to moderate the present effects of moralized rationality. We would expect those who moralize rationality to primarily assign blame to individuals who act irrationally if they are thought to have high (vs. low) capacity for rational thought. Thus, various factors that are associated with access to the resources (e.g., geographical location, education, power, and status), and with the cognitive capacity needed to form rational beliefs (e.g., intelligence, maturity) are plausible moderators of the present findings.

Another remaining question is exactly *why* individuals who moralize rationality perceive decisions and actions that stem from an irrational process as less moral than decisions and actions that stem from a rational process. According to many theories in moral psychology concerns about harm are at the core of moral considerations ([35, 36, 94]; but see [4, 95]). One possibility is therefore that the present findings were driven by concerns about harm. Specifically, individuals who moralize rationality may have reacted with moral condemnation towards irrational targets because their behavior was considered harmful. For example, the target person who relied on homeopathy (vs. medicine) to treat his medical condition may have been perceived as less moral because his behavior was perceived as harmful to himself and those who care about him.

We examined the role of harm in three different ways in the present studies, and we have no evidence to suggest that the moral concerns of those who moralize rationality are driven by harm considerations. First, we examined the relationship between the MRS and the Care/harm foundation in five independent samples (Study 1, Study 3, Study 6, Study 7, and Study 8). In all five studies, scores on the MRS were unrelated to subjective concerns about harm, including motivation to contribute to a charity tailored to capture the harm foundation (Study 8). Second, we investigated how the MRS related to utilitarian and deontological harm considerations in moral dilemmas (Study 5). Moralized rationality was independent of utilitarian harm considerations, and associated with an inclination to *reject* deontological harm considerations. Finally, in two out of four scenarios examined in Studies 6 and 7 (astrology, homeopathy) we experimentally controlled for harm by stating explicitly that the outcome of the irrational (as well as the rational) act was favorable. Thus, moral reactions to the irrational (vs. rational) act cannot be attributed to any objective harm in these scenarios, and we observed no differences in results in the two scenarios where harm could be implied (creationism, religious doctor). That said, however, we cannot rule out the possibility that the effects obtained were driven by more specific concerns about harm. In particular, it is still possible that individuals high in MR perceive, perhaps implicitly, that irrationally based acts are more harmful than acts rooted in reason [73, 96], or that their moral condemnation of irrational targets was based on counterfactual thinking (e.g., what if the cough *had* been a symptom of a serious disease?).

A second possible explanation is that moralized rationality could be viewed as an intellectual version of purity concerns. Individuals who score high on the MRS may respond to violations of rationality much in the same way as individuals who score high on purity respond to “unnatural” sexual acts [46]. Specifically, “intellectual purists” (i.e., those high in MR) may view irrational processes of forming and evaluating beliefs as immoral (and disgusting, as in Study 7) in and of themselves, regardless of their consequences.

A third possibility is that individuals high in MR recognize that it can be painful to examine one’s own beliefs in the light of reason and available evidence, because it may reveal that cherished beliefs are unfounded. This realization may cause people high in MR to view individuals who carefully scrutinize the foundations of their beliefs as courageous, and as possessing a strong character. By contrast, those high in MR may view individuals who are unwilling to examine their beliefs in the light of reason and evidence as cowardly, and as possessing a weak character.

Additional research is needed to determine whether the present findings were driven by condemnation of irrationality itself, or by condemnation of irrationality because it is perceived as harmful. Resolving this issue will not be an easy task, however. For example, people who score high on the MRS may readily come up with intuitive post-hoc explanations about how an irrationally formed position can cause harm, yet these harm concerns may have had little or no influence on their moral judgment [46]. Indeed, this is precisely why we decided to experimentally control for objective harm in the scenarios, and to measure more general harm concerns (Care/harm, Utilitarianism), rather than explicitly ask participants how much harm they perceived in the scenarios themselves. These methodological challenges aside, identifying exactly what it is about irrationality that makes individuals high in moralized rationality tick remains an important question for future research.

## Conclusion

As illustrated by the quotes from Thomas Jefferson and Charles Sanders Peirce at the beginning of this article, some people view it as a moral virtue to rely on reason and evidence when forming and evaluating beliefs. We have demonstrated that individual differences in the inclination to moralize rationality are stable over time, and cannot be reduced to any of the central moral values identified in the literature. Moralized rationality is not only related to the rejection of traditional beliefs that are not backed up by logic and evidence, but also leads to intolerance of those who endorse such beliefs, and motivation to support activist groups that work to prevent the spread of irrational beliefs. Moralized rationality may therefore play an important role in various conflicts that concern the validity of specific beliefs. Because moralized rationality centers on the appropriate processes of evaluating beliefs, rather than on their specific contents, it may also be a safeguard against motivated reasoning biases. It therefore strikes us as a fruitful enterprise to further examine the implications and antecedents of individual differences in moralized rationality.

## Supporting Information

S1 File Study 1 data. (SAV)

S2 File Study 2 data. (SAV)

S3 File Study 3 data. (SAV)

S4 File Study 4 data. (SAV)

S5 File Study 5 data. (SAV)

S6 File Study 6 data. (SAV)

S7 File Study 7 data. (SAV)

S8 File Study 8 data. (SAV)

S1 Text List of all MR and IR items in Study 1. Items included in the final MR and IR scales in italics.
(DOCX)

S2 Text Scenarios used to manipulate target rationality in Study 6. (DOCX)

S3 Text Scenarios used to manipulate target rationality in Study 7. (DOCX)

S4 Text Scenarios used to describe charities in Study 8. (DOCX)

S5 Text List of all variables in each dataset. (DOCX)
