# Supplementary material for: Moralized Rationality: Relying on Logic and Evidence in the Formation and Evaluation of Belief Can Be Seen as a Moral Issue
Source: PLoS One. 2016 Nov 16;11(11):e0166332. doi: 10.1371/journal.pone.0166332 (PMC5112873; doi:10.1371/journal.pone.0166332)
Supplement: S1 Text — Items included in the final MR and IR scales in italics. (DOCX) [file pone.0166332.s009.docx]

**S1 Text**

Items used to measure moralized rationality and Importance of rationality (Study 1)

Items included in the final MR and IR-scales are italicized

**Moralized Rationality**

1. Not being intellectually honest when evaluating the strength of one’s own arguments is immoral.

*2. Being skeptical about claims that are not backed up by evidence is a moral virtue.*

*3. Holding on to beliefs when there is substantial evidence against them is immoral.*

4. It is morally wrong to uncritically rely on someone’s authority when forming a belief about something.

5. To remain rational and levelheaded even in heated arguments is a moral virtue.

6. It is morally virtuous to examine traditionally held beliefs using logic and evidence.

*7. It is morally wrong to trust your intuitions without rationally examining them.*

*8. It is morally wrong to rely on anything else other than logic and evidence when deciding what is true and what is not true.*

9. Relying on logic and evidence when forming beliefs, rather than on tradition, is a moral virtue.

*10. It is a moral imperative that people can justify their beliefs using rational arguments and evidence.*

11. Doubting the results of scientific research because when they go against your personal beliefs and values is morally wrong.

*12. It is immoral to hold irrational beliefs.*

13. Critically examining one’s long-held beliefs shows moral courage.

*14. A person’s moral authority depends on their rationality.*

15. When people tenaciously hold on to false beliefs it says nothing about their morality. (R)

16. When deciding whether something is true or not, it is morally wrong to rely on an authority without critically examining that authority’s claims.

17. Whether a person’s core beliefs are rational or not says nothing about their morality (R).

18. I find it morally defensible to disagree with scientific findings based on personal beliefs. (R).

*19. A person’s morality is in no way determined by their rationality (R).*

20. Whether opinions are rational or not says nothing about their morality (R).

*21. Whether a person can be convinced by reason and evidence is in no way indicative of their morality (R).*

22. People who value rationality and evidence are just as moral as people who do not value rationality and evidence (R).

**Importance of Rationality**

1. It is not important to me personally to be intellectually honest when evaluating the strength of my own arguments (R).

*2. It is important to me personally to be skeptical about claims that are not backed up by evidence.*

3. It is important to me personally that I do not hold on to beliefs when there is substantial evidence against them.

4. It is important to me personally not to uncritically rely on someone’s authority when forming a belief about something.

*5. It is important to me personally to remain rational and levelheaded even in heated arguments.*

*6. It is important to me personally to examine traditionally held beliefs using logic and evidence.*

7. It is important to me personally not to trust my intuitions without rationally examining them.

8. It is important to me personally not to rely on anything else other than logic and evidence when deciding what is true and what is not true.

9. It is important to me personally to rely on logic and evidence when forming beliefs, rather than on tradition.

*10. It is important to me personally that I can justify my beliefs using rational arguments and evidence.*

11. It is important to me personally not to doubt the results of scientific research because even if they go against my personal beliefs and values.

12. It is important to me personally not to hold irrational beliefs.

*13. It is important to me personally to critically examine my long-held beliefs.*

*14. It is important to me personally to be a rational person.*

15. Whether I tenaciously hold on to false beliefs or not is unimportant to me personally (R).

16. When deciding whether something is true or not, it is important to me personally not to rely on an authority without critically examining that authority’s claims.

17. It is not important to me personally whether my core beliefs are rational or not (R).

18. It is not important to me personally whether scientific findings conflict with my personal beliefs (R).

19. Being a rational person is not important to me personally (R).

20. Whether my opinions are rational or not is not important to me personally (R).

21. It is not important to me personally whether the arguments someone uses to convince me of something are rational or not (R).

22. Rationality and evidence are not important to me (R).
