# Supplementary material for: Moralized Rationality: Relying on Logic and Evidence in the Formation and Evaluation of Belief Can Be Seen as a Moral Issue
Source: PLoS One. 2016 Nov 16;11(11):e0166332. doi: 10.1371/journal.pone.0166332 (PMC5112873; doi:10.1371/journal.pone.0166332)
Supplement: S2 Text — (DOCX) [file pone.0166332.s010.docx]

**S2 Text**

**Scenarios used to manipulate target rationality in three different domains (Study 6)**

Note: Because we suspected that participants would vary in their knowledge about homeopathy, we started by providing participants in this condition with some background information about homeopathy. Specifically, we informed participants about the basic principles of homeopathic remedies, about their widespread use in the western world, and about the fact that many people attest to their effectiveness in treating various medical conditions. We also informed participants that the scientific community has remained very skeptical about homeopathy, in part because the extreme dilution process used frequently results in a solution that does not contain a single molecule of the active ingredient, and also because the empirical evidence suggests that homeopathic remedies have no reliable effects. Because we assumed that people would be sufficiently familiar with both astrology and creationism for our purposes, no background information was provided in these conditions.

**Homeopathy**

*Irrational condition*

Over the last week, John has suffered from a cough. He eventually decided to go to his homeopath to see what he would recommend. The homeopath prescribed a homeopathic remedy for his cough. John started taking the remedy the very same day, and continued to use it over the next few days, as the homeopath had recommended. John soon started to feel better, and within a few days, his cough was completely gone. This experience strengthened John’s belief that it is a good idea to visit the homeopath whenever he is feeling unwell.

*Rational condition*

Over the last week, John has suffered from a cough. He eventually decided to go to his doctor to see what he would recommend. The doctor prescribed a medicine for his cough. John started taking the medicine the very same day, and continued to use it over the next few days, as the doctor had recommended. John soon started to feel better, and within a few days, his cough was completely gone. This experience strengthened John’s belief that it is a good idea to visit the doctor whenever he is feeling unwell.

**Astrology**

*Irrational condition*

John has saved some money over the years, and he is now considering investing his savings in stocks. The local newspaper recently mentioned that a new computer company – Aries – is going on the stock market. He thinks that the company’s name has a nice ring to it – because Aries is his astrological sign. Furthermore, his most recent horoscope informed him that this is a period during which he is likely to make beneficial financial decisions. John therefore decides to invest his savings in Aries stocks. A year later, John’s investment has doubled in size. He was very pleased that he trusted his instincts – as well as the recommendations from his horoscope. This experience solidified his belief that astrology forms a reliable path to making better life decisions.

*Rational condition*

John has saved up some money over the years, and he is now considering investing his savings in stocks. The local newspaper recently mentioned that a new computer company – Aries – is going on the stock market. He starts looking into the company in great detail. He carefully examines Aries’ financial situation, the business plan, and he also talks to several friends who work as stockbrokers and IT professionals. All the information he gathers indicates that Aries is a very promising company, and that chances are high that their stock is going to go up substantially. John therefore decides to invest his savings in Aries stocks. A year later, John’s investment has doubled in size. He was very pleased that he trusted his own careful analysis of the company – as well as the recommendations from his friends with relevant expertise. This experience solidified his belief that careful analysis of relevant facts forms a reliable path to making better life decisions.

**Creationism**

*Irrational condition*

John is very skeptical about the theory of evolution. His position is that the theory has a lot of holes in it that haven’t been addressed. In his own words: “If the theory of evolution was true, you would expect to find a lot more ‘intermediary life forms’ in the fossil record – but they aren’t there. Furthermore, according to the Bible, God created the world - and everything in it - in 7 days. That includes us humans. So, the idea that humans evolved over millions of years from much simpler biological beginnings is simply inconsistent with the Bible’s account of creation. Since the Bible is the infallible word of God, the theory of evolution must be wrong.

*Rational condition*

John is convinced that the theory of evolution is true. His position is that the evidence in support of the theory is massive, and that people who suggest that the theory has a lot of holes in it don’t understand enough about science. In his own words: “We can now trace the origin of species in great detail through the fossil record. Through our advancements in molecular biology, we can also explain how the process works at the genetic level. Opponents of the theory of evolution either do not understand this evidence, or choose to ignore it. And by the way, what is their alternative explanation? That God did it. Well that idea has absolutely no evidence in its favor. Since there is so much evidence for evolution, the creation story from the Bible is almost certainly wrong.
