# Supplementary material for: Moralized Rationality: Relying on Logic and Evidence in the Formation and Evaluation of Belief Can Be Seen as a Moral Issue
Source: PLoS One. 2016 Nov 16;11(11):e0166332. doi: 10.1371/journal.pone.0166332 (PMC5112873; doi:10.1371/journal.pone.0166332)
Supplement: S3 Text — (DOCX) [file pone.0166332.s011.docx]

**S3 Text**

**Scenarios used to manipulate target rationality (Study 7)**

*Irrational condition: Doctor acting on religious faith*

Richard is a general practitioner with his own practice in a small town in South Carolina. He is often visited by Mary, who suffers from diffuse symptoms such as tiredness, headaches and muscle fatigue. Despite running numerous tests, Richard can find nothing wrong with Mary, and can therefore not diagnose her with an illness.

Instead, Richard advises the devoutly Christian Mary to pray to God for improvement of her health, and to come back in two weeks if her symptoms remained unchanged. Richard believes that God listens to the prayers of those who have faith and lead righteous lives, as it is said in the Holy Bible. Because Mary is such a faithful Christian, Richard reasons, advising her to pray to God for good health may just help alleviate some of her symptoms. Thus, because the Bible says that God listens to the prayers of the faithful, Richard believes that praying to God may help Mary feel better.

*Rational condition: Doctor acting on evidence*

Richard is a general practitioner with his own practice in a small town in South Carolina. He is often visited by Mary, who suffers from diffuse symptoms such as tiredness, headaches and muscle fatigue. Despite running numerous tests, Richard can find nothing wrong with Mary, and can therefore not diagnose her with an illness.

Instead, Richard advises the devoutly Christian Mary to pray to God for improvement of her health, and to come back in two weeks if her symptoms remained unchanged. Although he knows that praying to God in itself doesn’t help cure diseases, Richard is aware of the placebo effect; he knows that research shows that the mere expectation that a treatment will be effective can alleviate a patient’s symptoms. Because Mary is such a faithful Christian, Richard reasons, advising her to pray to God for good health may just help alleviate some of her symptoms. Thus, based on research on the placebo effect, Richard believes that praying to God may help Mary feel better.
