# Supplementary material for: Moralized Rationality: Relying on Logic and Evidence in the Formation and Evaluation of Belief Can Be Seen as a Moral Issue
Source: PLoS One. 2016 Nov 16;11(11):e0166332. doi: 10.1371/journal.pone.0166332 (PMC5112873; doi:10.1371/journal.pone.0166332)
Supplement: S4 Text — (DOCX) [file pone.0166332.s012.docx]

**S4 Text**

**The text used to describe the six charities in Study 8.**

In this section of the survey we would like to hear your opinion about different kinds of charities. You will be presented with descriptions of six different charities, each working for a different cause. You will be asked some questions about your attitude towards each of the charities, and your willingness to support their causes. The charities that will be described here do not exist in real life. For the purpose of this study, however, we ask you to imagine that the charities described actually do exist.

*Skeptic Alliance* (Moralized Rationality)

Skeptic Alliance is an American non-profit organization devoted to preventing the spread of pseudoscience, superstition, and other kinds of irrational beliefs. Skeptic Alliance funds scientific studies that put widespread superstitious ideas to the test. Skeptic Alliance also organizes lecture series on critical thinking and scientific skepticism, and publishes a quarterly magazine that debunks common myths and superstitions.

Skeptic Alliance would thus use your donation to fund research and outreach activities designed to prevent the spread of various irrational beliefs.

*Project Compassion* (Care/harm)

Project Compassion is an American non-profit organization devoted to helping mistreated animals. Project Compassion sends teams of veterinarians to find and help domesticated and farm animals that are mistreated by their owners. Project Compassion is dedicated to finding such animals, removing them from their abusive environment, curing them, and finding them a good new home.

Project Compassion would thus use your donation to seek out and help mistreated animals, and to find them better homes.

*Justice for All* (Fairness/cheating)

Justice for All is an American non-profit organization devoted to providing legal assistance to people that cannot afford it themselves. Justice for All sends volunteer legal professionals to seek out and help individuals who live in poverty and who lack the financial means to defend themselves in the judicial system.

Justice for All would thus use your donation to give legal assistance to people who live in poverty.

*In the Line of Duty* (Authority/subversion)

In the Line of Duty is an American non-profit organization devoted to supporting the families of former police officers that have been killed or injured in the line of duty. The non-profit provides legal aid for the families and funds physical as well as psychological therapy.

In the Line of Duty would thus use your money to provide support for the families of police officers who have been injured or killed on the job.

*Giving back to our Heroes* (Loyalty/betrayal)

Giving back to our Heroes is an American non-profit organization devoted to supporting American war veterans. Giving back to our Heroes supports veterans by helping them reintegrate into society after they have served our nation abroad. In addition, they fund physical and psychological therapy, as well as legal counseling for veterans in need.

Giving back to our Heroes would thus use your donation to provide assistance to American servicemen returning from deployment abroad.

*Worth the Wait* (Sanctity/degradation)

Worth the Wait is a non-profit organization devoted to the promotion of sexual abstinence until marriage. This non-profit organizes lecture series and workshops for teenagers and their parents. It also provides age-appropriate educational materials on the topic of sexual abstinence.

Worth the Wait would thus use your donation to promote sexual abstinence until marriage among teenagers.
