# Supplementary material for: Moralized Rationality: Relying on Logic and Evidence in the Formation and Evaluation of Belief Can Be Seen as a Moral Issue
Source: PLoS One. 2016 Nov 16;11(11):e0166332. doi: 10.1371/journal.pone.0166332 (PMC5112873; doi:10.1371/journal.pone.0166332)
Supplement: S5 Text — (DOCX) [file pone.0166332.s013.docx]

**Study 1 Variable list**

Harm 1-6 = Care/Harm items (MFQ)

Fair 1-6 = Fairness/Cheating items (MFQ)

Loyal 1-6 = Loyalty/betrayal items (MFQ)

Authority 1-6 = Authority/subversion items (MFQ)

Purity 1-6 = Sanctity/degradation items (MFQ)

Liberty 1-9 = Liberty/oppression items (MFQ)

IR 1-22 = Importance of rationality items

MR 1-22 = Moralized rationality items

Gender = participant gender

Age = participant age

Race = race/ethnicity

Education = level of education

PoliticalSocial = Political orientation (social issues)

PoliticalEco = Political orientation (economic issues)

SC 1-3 = Religiosity items (Santa Clara)

Relpref = Religious preference

Jewish_den = Jewish denominations

Christian_den = Christian denominations

Other_Rel = Other religious denominations

Non_Rel = Nonreligious orientations

Filter = Duplicate IP addresses and extremely fast responders

*Items with an “R” at the end are reverse-coded

**Study 2 Variable list**

MR 1-10 = Moralized rationality items

IR 1-7 = Importance of rationality items

Gender = participant gender

Age = participant age

Race = race/ethnicity

Education = level of education

PoliticalSocial = Political orientation (social issues)

PoliticalEco = Political orientation (economic issues)

Relpref = Religious preference

Jewish_den = Jewish denominations

Christian_den = Christian denominations

Other_Rel = Other religious denominations

Non_Rel = Nonreligious orientations

*Items with an “R” at the end are reverse-coded

**Study 3 Variable list**

Harm 1-6 = Care/Harm items (MFQ)

Fairness 1-6 = Fairness/Cheating items (MFQ)

Loyalty 1-6 = Loyalty/betrayal items (MFQ)

Authority 1-6 = Authority/subversion items (MFQ)

Purity 1-6 = Sanctity/degradation items (MFQ)

Liberty 1-9 = Liberty/oppression items (MFQ)

MR 1-9 = Moralized rationality items

IR 1-6 = Importance of rationality items

Gender = participant gender

Age = participant age

Race = race/ethnicity

Education = level of education

PoliticalSocial = Political orientation (social issues)

PoliticalEco = Political orientation (economic issues)

SC 1-3 = Religiosity (Santa Clara)

Relpref = Religious preference

Non_Rel = Nonreligious orientations

Other_Rel = Other religious denominations

Jewish_Den = Jewish denominations

Christian_Den = Christian denominations

Filter = Duplicate IP addresses and extremely fast responders

*Items with an “R” at the end are reverse-coded

**Study 4 Variable list**

Study = Recruited from Study 1 (4 months earlier) or Study 6 (2 months earlier)

MR 1-9 = Moralized Rationality items (Time 1)

IR 1-6 = Importance of Rationality items (Time 1)

MR 1-9T2 = Moralized Rationality items (Time 2)

IR 1-6T2 = Importance of Rationality items (Time 2)

Gender = participant gender

Age = participant age

Race = race/ethnicity

Education = level of education

SC 1-3 = Religiosity (Santa Clara)

MR8Rec = Moralized Rationality item 8 Recoded (Time 1)

MR9Rec = Moralized Rationality item 9 Recoded (Time 1)

MR8T2Rec = Moralized Rationality item 8 Recoded (Time 2)

MR9T2Rec = Moralized Rationality item 9 Recoded (Time 2)

MRS = Moralized Rationality Scale (Time 1)

IRS = Importance of Rationality Scale (Time 1)

MRST2 = Moralized Rationality Scale (Time 2)

IRST2 = Importance of Rationality Scale (Time 2)

*Variables with an “R” at the end are reverse-coded (e.g., MR8R)

**Study 5 Variable list**

Para 1-6 = Paranormal beliefs items

TradRoleModel 1-9 = Prototypicality of Traditional Moral Traits (for amoral role model)

RatRoleModel 1-4 = Prototypicality of Rationality for Morality (for a moral role model)

TradImmoral 1-9 = Prototypicality of Traditional Immoral Traits (Immoral person)

RatImmoral 1-4 = Prototypicality of Irrational Traits (Immoral person)

BiS 1-9 = Belief in Science items

MR 1-9 = Moralized Rationality items

IR 1-6 = Importance of Rationality items

SDE 1-20 = Self-Deceptive Enhancement items

IM 1-20 = Impression Management items

Gender = participant gender – ADD values

Age = participant age

Race = race/ethnicity – ADD values

Education = level of education

PoliticalSocial = Political orientation (social issues)

PoliticalEco = Political orientation (economic issues)

SC 1-3 = Religiosity (Santa Clara)

Relpref = Religious preference

Non_Rel = Nonreligious orientations

Other_Rel = Other religious denominations

Jewish_Den = Jewish denominations

Christian_Den = Christian denominations

SDEScore 1-20 = Score on each SDE item (1 or 0)

IMScore 1-20 = Score on each IM item (1 or 0)

SDEscale = Self-Deceptive Enhancement scale (Total)

IMscale = Impression Management scale (Total)

Incongruent 1-10 = Response to incongruent moral dilemmas

Congruent 1-10 = Response to congruent moral dilemmas

CongruentTotal = Number of “Inappropriate” responses to congruent dilemmas

IncongruentTotal = Number of “Inappropriate” responses to incongruent dilemmas

CongruentProportion = CongruentTotal / 10

IncongruentProportion = IncongruentTotal / 10

Utilitarianism = Utilitarian inclinations (process dissociation; for details, see Conway & Gawronski, 2013)

Deontology = Deontological inclinations (process dissociation; for details, see Conway & Gawronski, 2013)

MR8Rec = Moralized Rationality item 8 Recoded

MR9Rec = Moralized Rationality item 9 Recoded

MRS = Moralized Rationality Scale

IRS = Importance of Rationality Scale

ParanormalBeliefs = Paranormal Beliefs Scale

BIS = Belief in Science Scale

ProtRationalityMorality = Prototypicality of Rationality for Morality Scale

ProtTradMoralTraits = Prototypicality of Trational Moral Traits Scale

*Items with an “R” at the end are reverse-coded

**Study 6 Variable list**

MR 1-9 = Moralized Rationality items

IR 1-6 = Importance of Rationality items

Harm 1-6 = Care/Harm items (MFQ)

Fairness 1-6 = Fairness/Cheating items (MFQ)

Loyalty 1-6 = Loyalty/betrayal items (MFQ)

Authority 1-6 = Authority/subversion items (MFQ)

Purity 1-6 = Sanctity/degradation items (MFQ)

Liberty 1-9 = Liberty/oppression items (MFQ)

Predist 1-6 = Baseline social distance items

Postdist 1-6 = Post target rationality manipulation social distance items

Competent = Target competence item 1

Rational = Target competence item 2

Warm = Target warmth item 1

Friendly = Target warmth item 2

Good = Target morality item 1

Moral = Target morality item 2

Ethical = Target morality item 3

Gender = participant gender

Age = participant age

Race = race/ethnicity

Education = level of education

PoliticalSocial = Political orientation (social issues)

PoliticalEco = Political orientation (economic issues)

SC 1-3 = Religiosity (Santa Clara)

Relpref = Religious preference

Non_Rel = Nonreligious orientations

Other_Rel = Other religious denominations

Jewish_Den = Jewish denominations

Christian_Den = Christian denominations

Domain = Manipulation of domain

Rationality = Manipulation of target rationality

PraiseJudgment = Perceived praiseworthiness of the target’s action

MoralJudgment = Perceived morality of the target’s action

BlameJudgment = Perceived blameworthiness of the target’s action

ImmoralJudgment = Perceived immorality of the target’s action

HarmScale = Care/Harm Foundation

FairnessScale = Fairness/Cheating Foundation

LoyaltyScale = Loyalty/Betrayal Foundation

AuthorityScale = Authority/Subversion Foundation

PurityScale = Sanctity/degradation Foundation

LibertyScale = Liberty/Oppression Foundation

MR8Rec = MRS item 8 recoded (original reverse-coded)

MR9Rec = MRS item 9 recoded (original reverse-coded)

MRS = Moralized Rationality Scale

IRS = Importance of Rationality Scale

Predistance = Baseline social distance scale

Postdistance = Post-manipulation social distance scale

Approach = Postdistance – Predistance (score above 0 = approach; score below 0 = distancing)

CompetenceScale = Target competence scale (Competent & Rational)

WarmthScale = Target warmth scale (Warm & Friendly)

MoralityScale = Target morality scale (Good, Moral, & Ethical)

ZMRS = Standardized Moralized Rationality scores (Z-scores)

ZIRS – Standardized Importance of Rationality scores (Z-scores)

Filter = Duplicate IP-addresses and extremely fast responders

ImmoralJudgmentRec = ImmoralJudgment Recoded

MoralJudgmentScale = MoralJudgment and ImmoralJudgmentRec

RationalityEffectCoded = Target rationality manipulation (effect-coded)

RationalityXMRS = Target rationality by MRS interaction term

HighMRS = 1 SD above the mean (for simple slope analysis)

LowMRS = 1 SD below the mean (for simple slope analysis)

RationalityXLowMRS = Interaction term for simple slope analysis

RationalityXHighMRS = Interaction term for simple slope analysis

Distancing = Approach recoded (score above 0 = distancing; score below 0 = approach)

PraiseRec = PraiseJudgment recoded

BlamePraiseScale = BlameJudgment & PraiseRec

*Items with an “R” at the end are reverse-coded

**Study 7 variable list**

MR 1-9 = Moralized Rationality items

IR 1-6 = Importance of Rationality items

Harm 1-6 = Care/Harm items (MFQ)

Fairness 1-6 = Fairness/Cheating items (MFQ)

Loyalty 1-6 = Loyalty/betrayal items (MFQ)

Authority 1-6 = Authority/subversion items (MFQ)

Purity 1-6 = Sanctity/degradation items (MFQ)

Liberty 1-9 = Liberty/oppression items (MFQ)

Punish 1-6 = Desire for punishment of target items

Anger = Anger towards target

Disgust = Disgust towards target

Contempt = Contempt towards target

Gender = participant gender

Age = participant age

Race = participant race/ethnicity

Education = participant level of education

PoliticalSocial = Political orientation (social issues)

PoliticalEco = Political orientation (economic issues)

SC 1-3 = Religiosity (Santa Clara)

Relpref = Religious preference

Non_Rel = Nonreligious orientations

Other_Rel = Other religious denominations

Jewish_Den = Jewish denominations

Christian_Den = Christian denominations

Rationality = Manipulation of target rationality

Filter = Extremely fast responders

MR8Rec = MRS item 8 recoded (original reverse-coded)

MR9Rec = MRS item 9 recoded (original reverse-coded)

MRS = Moralized Rationality Scale

IRS = Importance of Rationality Scale

Liberty6Rec = Liberty/Oppression item 6 Recoded (original reverse-coded)

HarmScale = Care/Harm Foundation

FairnessScale = Fairness/Cheating Foundation

LoyaltyScale = Loyalty/Betrayal Foundation

AuthorityScale = Authority/Subversion Foundation

PurityScale = Sanctity/degradation Foundation

LibertyScale = Liberty/Oppression Foundation

Punish4Rec = Desire for punishment item 4 recoded (original reverse-coded)

Punish5Rec = Desire for punishment item 5 recoded (original reverse-coded)

PunishmentScale = Desire for punishment of target scale

MoralEmotionsScale = Negative moral emotions towards target (anger, disgust, contempt)

RationalityEffectCoded = Manipulation of Target rationality effect-coded

ZMRS = Standardized Moralized Rationality scores (Z-scores)

ZIRS = Standardized Importance of Rationality scores (Z-scores)

RationalityXMRS = Target rationality by MRS interaction term

RationalityXIRS = Target rationality by IRS interaction term

LowMRS = 1 SD below the Mean (for simple slope analysis)

HighMRS = 1 SD above the Mean (for simple slope analysis)

RationalityXLowMRS = Interaction term for simple slope analysis

RationalityXHighMRS = Interaction term for simple slope analysis

*Items with an “R” at the end are reverse-coded

**Study 8 Variable list**

MR 1-9 = Moralized Rationality items

IR 1-6 = Importance of Rationality items

Harm 1-6 = Care/Harm items (MFQ)

Fairness 1-6 = Fairness/Cheating items (MFQ)

Loyalty 1-6 = Loyalty/betrayal items (MFQ)

Authority 1-6 = Authority/subversion items (MFQ)

Purity 1-6 = Sanctity/degradation items (MFQ)

SkepticVolunteer = Willingness to volunteer for Skeptic Alliance

SkepticDonate = Willingness to donate to Skeptic Alliance

CompassionVolunteer = Willingness to volunteer for Project Compassion

CompassionDonate = Willingness to donate to Project Compassion

JusticeVolunteer = Willingness to volunteer for Justice for All

JusticeDonate = Willingness to donate to Justice for All

DutyVolunteer = Willingness to volunteer for In the Line of Duty

DutyDonate = Willingness to donate to In the Line of Duty

HeroesVolunteer = Willingness to volunteer for Giving Back to our Heroes

HeroesDonate = Willingness to donate to Giving Back to our Heroes

WaitVolunteer = Willingness to volunteer for Worth the Wait

WaitDonate = Willingness to donate to Worth the Wait

Donations_Skeptic = Zero-sum donations to Skeptic Alliance (out of $50)

Donations_Compassion = Zero-sum donations to Project Compassion (out of $50)

Donations_Justice = Zero-sum donations to Justice for All (out of $50)

Donations_Duty = Zero-sum donations to In the Line of Duty (out of $50)

Donations_Heroes = Zero-sum donations to Giving back to our Heroes (out of $50)

Donations_Wait = Zero-sum donations to Worth the Wait (out of $50)

Gender = participant gender

Age = participant age

Race = race/ethnicity

Education = level of education

PoliticalSocial = Political orientation (social issues)

PoliticalEco = Political orientation (economic issues)

SC 1-3 = Religiosity (Santa Clara)

Relpref = Religious preference

Non_Rel = Nonreligious orientations

Other_Rel = Other religious denominations

Jewish_Den = Jewish denominations

Christian_Den = Christian denominations

MR8Rec = MRS item 8 recoded (original reverse-coded)

MR9Rec = MRS item 9 recoded (original reverse-coded)

MRS = Moralized Rationality Scale

IRS = Importance of Rationality Scale

HarmScale = Care/Harm Foundation

FairnessScale = Fairness/Cheating Foundation

LoyaltyScale = Loyalty/Betrayal Foundation

AuthorityScale = Authority/Subversion Foundation

PurityScale = Sanctity/degradation Foundation

*Items with an “R” at the end are reverse-coded
